# A ten-genes-based diagnostic signature for atherosclerosis

**DOI:** 10.1186/s12872-021-02323-9

**Published:** 2021-10-23

**Authors:** Feng Zhu, Lili Zuo, Rui Hu, Jin Wang, Zhihua Yang, Xin Qi, Limin Feng

**Affiliations:** 1 Graduate School, Tianjin University of Traditional Chinese Medicine, Tianjin, China; 2 Department of Traditional Chinese Medicine, Hebei North University, Zhangjiakou City, Hebei Province China; 3 Department of Cardiology, Tianjin Union Medical Center, Tianjin, China; 4 Department of Neonatal, ZiBo Maternal and Child Health Hospital, Zibo City, Shandong Province China; 5 Center for Drug Monitoring and Evaluation Department, Center for Drug Monitoring and Evaluation in Zhangjiakou, Zhangjiakou City, Hebei Province China; 6 Department of Cardiovascular Disease, ZiBo Hospital of Traditional Chinese Medicine, Zibo City, Shandong Province China; 7 First Teaching Hospital of Tianjin University of Traditional Chinese Medicine, Tianjin, China; 8 Department of Cardiology, The Second Affiliated Hospital of Tianjin University of Traditional Chinese Medicine, Tianjin, China

**Keywords:** Atherosclerosis, GO analysis, KEGG analysis, PPI network, Logistic regression diagnostic mode

## Abstract

**Background:**

Atherosclerosis is the leading cause of cardiovascular disease with a high mortality worldwide. Understanding the atherosclerosis pathogenesis and identification of efficient diagnostic signatures remain major problems of modern medicine. This study aims to screen the potential diagnostic genes for atherosclerosis.

**Methods:**

We downloaded the gene chip data of 135 peripheral blood samples, including 57 samples with atherosclerosis and 78 healthy subjects from GEO database (Accession Number: GSE20129). The weighted gene co-expression network analysis was applied to identify atherosclerosis-related genes. Functional enrichment analysis was conducted by using the clusterProfiler R package. The interaction pairs of proteins encoded by atherosclerosis-related genes were screened using STRING database, and the interaction network was further optimized with the cytoHubba plug-in of Cytoscape software.

**Results:**

The logistic regression diagnostic model was constructed to predict normal and atherosclerosis samples. A gene module which included 532 genes related to the occurrence of atherosclerosis were screened. Functional enrichment analysis basing on the 532 genes identified 235 significantly enriched GO terms and 44 significantly enriched KEGG pathways. The top 50 hub genes of the protein–protein interaction network were identified. The final logistic regression diagnostic model was established by the optimal 10 key genes, which could distinguish atherosclerosis samples from normal samples.

**Conclusions:**

A predictive model based on 10 potential atherosclerosis-related genes was obtained, which should shed light on the diagnostic research of atherosclerosis.

**Supplementary Information:**

The online version contains supplementary material available at 10.1186/s12872-021-02323-9.

## Background

Cardiovascular disease (CVD) is the leading cause of mortality worldwide, and the underlying cause of the majority of CVD is atherosclerosis [1]. As a chronic vascular disease of the arterial wall, atherosclerosis is considered as a major cause of death and loss of productive life years in the world [2]. Atherosclerosis is an important pathological factor of cardiovascular disease (CVD), the leading killer in America [3]. In the last decades, atherosclerosis is considered as a predominantly lipid-driven disease, which is characterized by lipid deposition in the arterial wall [4]. Recently, a series of advances in the therapy of atherosclerosis have been achieved, for example, nanotechnology shows an encouraging prospect in the treatment of atherosclerosis via the functions of nanoagents, which kill the target cells after navigating in the blood, escaping from the biological barriers in the body, and assembling at the lesions [5]. Statin strategy is considered as an efficient therapeutic approach to combat atherosclerosis, and the combination therapy including statins and angiotensin II receptor blockers exhibits an synergistic anti-atherosclerotic effects during the occurrence and development of atherosclerosis [6, 7]. However, due to the non-obvious or asymptomatic features of the early symptoms of atherosclerosis [8], early diagnosis and intervention can efficiently prevent the disease from developing further, which is very necessary for the treatment of atherosclerosis.

With the progress of society and the development of technology, numerous risk factors in atherosclerosis have been identified and are considered as potential therapeutic strategies or diagnostic makers. Cholesterol acyltransferase (LCAT), the only enzyme which esterifies cholesterol in plasma and determines the maturation of high-density lipoproteins, could be modulated in a potential therapeutic strategy to reduce cardiovascular risk [9]. Wnt signaling is implicated in the progression of vascular lesions in various manners, involving in endothelial dysfunction, macrophage activation and the proliferation and migration of vascular smooth muscle cells, and might be considered as a promising therapeutic target for atherosclerosis [10]. Despite of these advances in the treatment, the identification of diagnostic makers is still urgent due to the lack of obvious symptoms in the early stage of atherosclerosis. Tibaut et al. has summarized researches on the serum biomarkers of atherosclerosis, of which, high sensitivity C-reactive protein (hsCRP) is considered as the most prospective biomarker in chronic situations [11]. In addition, microRNAs, which are non-coding and highly conserved small RNAs, have been reported to be novel biomarkers in the diagnosis and prognosis of atherosclerosis [12]. Although multiple risk factors in serum have been identified as diagnostic makers, more efficient diagnostic signatures in the peripheral blood of atherosclerosis patients and more reliable diagnostic models are needed to be explored in detail.

In this study, we identified 10 key genes which were predicted to be associated with the occurrence of atherosclerosis. Meanwhile, a diagnostic model was constructed, which might enrich the early diagnosis methods of atherosclerosis and has a certain practical value.

## Methods

### Datasets

We downloaded the gene chip data of 135 peripheral blood samples including 57 samples with atherosclerosis and 78 healthy subjects from GEO database [https://www.ncbi.nlm.nih.gov/geo/, Accession Number: GSE20129]. The coronary artery calcium (CAC) score was used to define atherosclerosis. The cases with CAC score > 100 were defined as atherosclerosis. Of which, the mRNA profiles of 119 samples were quantified by Illumina humanRef-8 v2.0 expression bead chip, and the mRNA profiles of the remaining 16 samples were quantified by Illumina HumanHT-12 V4.0 expression bead chip. Another dataset GSE43292 was also downloaded from GEO database, which consisted of 32 atherosclerotic plaque samples and 32 control samples. The samples in GSE43292 dataset were quantified by Affymetrix Human Gene 1.0 ST Array platform.

### Weighted gene co-expression network analysis

The WGCNA (Weighted Gene Co-expression Network Analysis) was performed by using “WGCNA” package of R language [13]. Firstly, the hierarchical clustering of genes was performed based on the gene expression values, the method of dynamic shear tree was used to identify gene modules and the genes with high similarity were grouped into the same module. Next, the Module Eigengene (ME) value of each module and the correlation coefficient between ME value and phenotype was calculated. Of which, phenotypes referred to disease states (dichotomous phenotypes), i.e., atherosclerosis and normal, here.

### Functional enrichment analysis

For the genes in the modules, Gene Ontology (GO) terms which consisted of biological process, molecular function and cellular component, and Kyoto Encyclopedia of Genes and Genomes (KEGG) pathway enrichment analysis were carried out by using clusterProfiler package of R language [14], and *p* value less than 0.05 was considered as statistically significant.

### Protein–protein interaction network

The STRING (https://string-db.org/,version 11.0) is a database for the analysis and prediction of functional connections and interactions of proteins [15]. Herein, STRING was used to analyze the functional connections and interactions between proteins. Then the visualization of the PPI network was achieved based on Cytoscape (https://cytoscape.org/, version 3.7.2) [16], with the key genes screened by the cytoHubba plug-in of Cytoscape software based on the Maximal Clique Centrality (MCC) algorithm.

### Logistic regression analysis

Logistic regression is a commonly used method in classification [17], which predicts a classification result based on a set of variables. In this study, the type of samples (whether the samples were atherosclerotic or not) was predicted with the expression value of mRNA as the variables. The 119 samples detected by Illumina humanRef-8 v2.0 expression bead chip were divided into the training set and the validation set for logistic model construction and validation based on the fivefold Cross Validation method, and then the remaining 16 samples were used as an independent validation set to further determine the reliability of the model. Specifically, according to the normal control samples and samples with atherosclerosis, Multivariate logistic regression model was constructed by using the glm function in R language [18]. The gene expression was adopted as the continuous variable and the sample type as the binary classification response value. The stepwise regression method was used to further screen the variables, and the model was reconstructed with the screened variables. Finally, the variable with p value less than 0.05 was used to establish the final model.

## Results

### WGCNA analysis

**Fig. 1:**
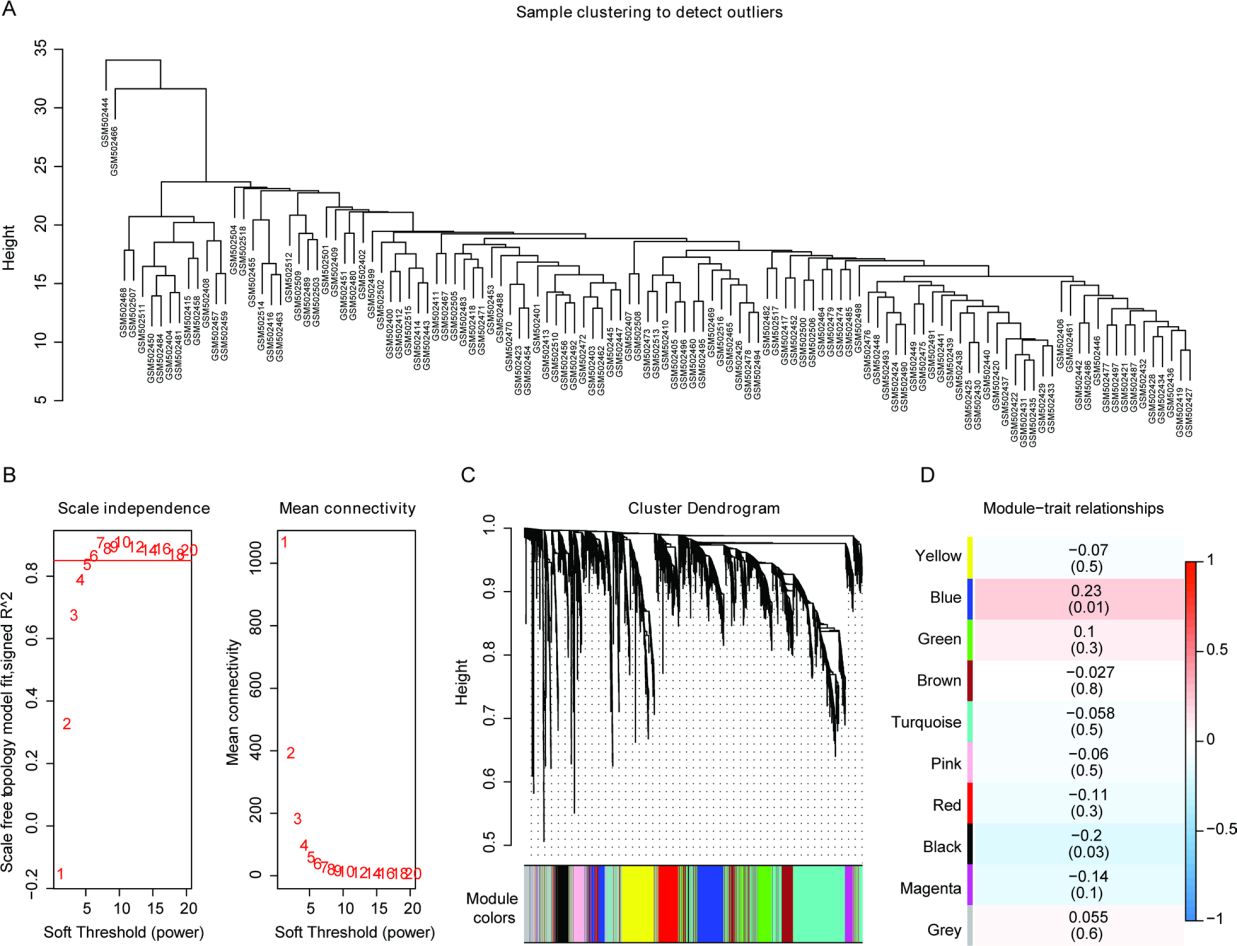
The WGCNA analysis. **A** Cluster analysis of 119 samples. **B** Selection of the soft threshold in the WGCNA analysis. The red line represents the correlation coefficient, and the first point above the red line corresponds to the soft threshold. **C** Clustering dendrogram of gene modules. Different gene modules are represented by distinct colors, and the genes that could not be grouped into other modules are placed in the grey module. **D** The correlation analysis between gene modules and phenotypes. The darker color indicates greater correlation between the gene module and the corresponding phenotype

We first performed the consensus clustering analysis with the mRNA profiles of 119 samples analyzed by Illumina humanRef-8 v2.0 expression bead chip platform (Fig. 1A), and the results revealed two outliers that were excluded in the subsequent analysis. Previous studies have indicated that the co-expression network conforms to the feature of unsigned network [19, 20], that is, log(k) (in which k represents the degree of a node) should be negatively correlated with log(P(k)) (P indicates the probability of the node appearance), and the correlation coefficient should be greater than 0.90. To ensure the unsigned characteristic of the co-expression network, the threshold β = 6 was selected (Fig. 1B). Next, the average-linkage hierarchical clustering method was used for the clustering of genes, and the minimum cardinality of each module was set as 100 according to the standard of mixed dynamic shear tree. Then the ME value of each module was calculated and cluster analysis of the modules was performed. Meanwhile, the modules close to each other were merged into a new one, with the height set as 0.25. A total of 10 modules were obtained, in which, the grey module was the genes that could not be grouped into other modules (Fig. 1C). Moreover, the correlation between each module and the sample type was calculated based on the ME value, and the blue module showed the greatest correlation with the sample type (Fig. 1D, correlation coefficient = 0.23 and *p* = 0.01). The genes in the blue module were shown in Additional file 2: Table S1. These results indicated that the genes in the blue module might be related to the initiation of atherosclerosis.

### GO term and KEGG pathway enrichment analysis

**Fig. 2:**
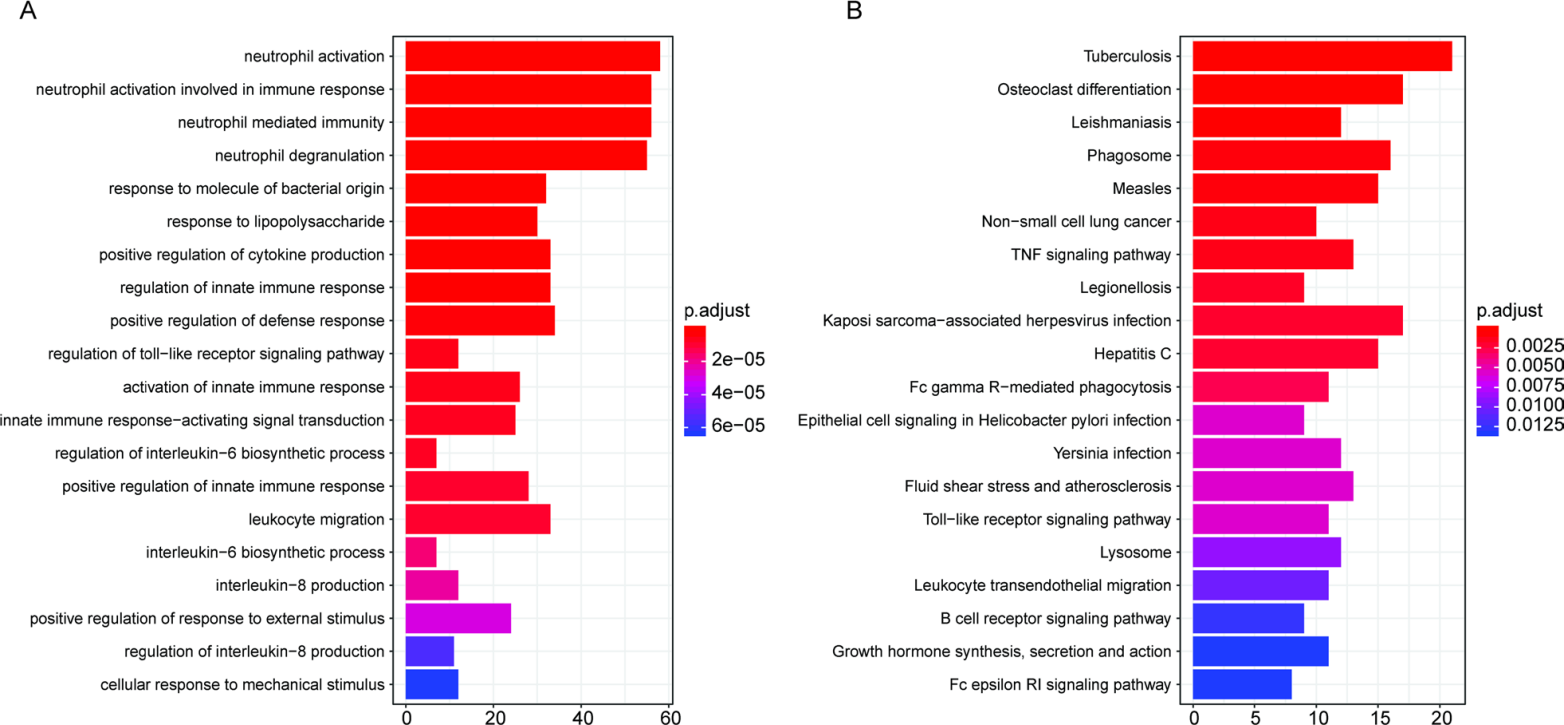
The GO and KEGG pathway enrichment analysis. **A** The top 20 GO terms with the greatest number of enriched genes. The horizontal axis denotes the gene number, and the vertical axis denotes the GO term. **B** The top 20 KEGG pathways with the greatest number of enriched genes. The horizontal axis denotes the gene number, and the vertical axis denotes the KEGG pathway

The GO term analysis and KEGG pathway enrichment analysis were performed based on the 532 genes in the blue module. The results showed that 235 GO terms (Additional file 3: Table S2) and 43 KEGG pathways were significantly enriched (Additional file 4: Table S3). Meanwhile, the top 20 most significantly enriched GO terms were shown in Fig. 2A, and the top 20 most significantly enriched KEGG pathways were shown in Fig. 2B. These results indicated that these genes which were predicted to be associated with atherosclerosis occurrence participated in various biological processes.

### Screening of hub genes in the PPI network

**Fig. 3:**
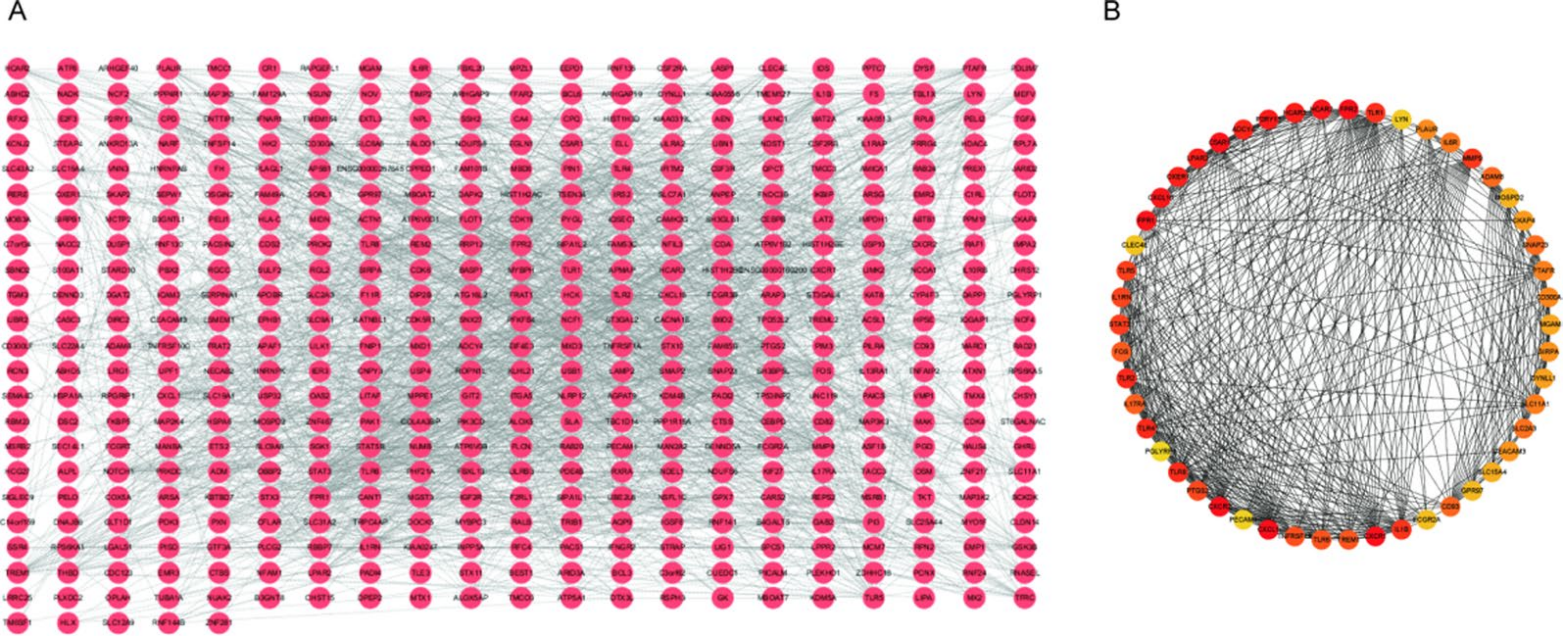
Construction of the PPI network. **A** The PPI network, in which the dot denotes the node. More lines connected to the dot indicates higher degree of this node and significant importance of the corresponding gene in the network. **B** The top 50 high-degree genes screened from the PPI network by using MCC algorithm. The darker the color is, the higher the degree is

The PPI network was constructed based on the 532 genes in the blue module using STRING database, and the interaction pairs with confidence score ≥ 0.4 were selected. Then the PPI network was visualized using the Cytoscape software (Fig. 3A). There were 467 nodes and 1920 edges in the PPI network, where the nodes denote genes and the edges denote the interactions between them. Then Cytoscape software was used to analyze the topological structure of the whole PPI network, and MCC algorithm was applied to score based on the importance of each node. The top 50 genes were screened according to the score (Fig. 3B), and the darker the color was, the more important the node was. And these 50 genes and corresponding scores were shown in Additional file 5: Table S4. These results indicated that the 50 genes might be associated with the occurrence of atherosclerosis.

### The logistic regression model for the diagnosis of atherosclerosis

**Fig. 4:**
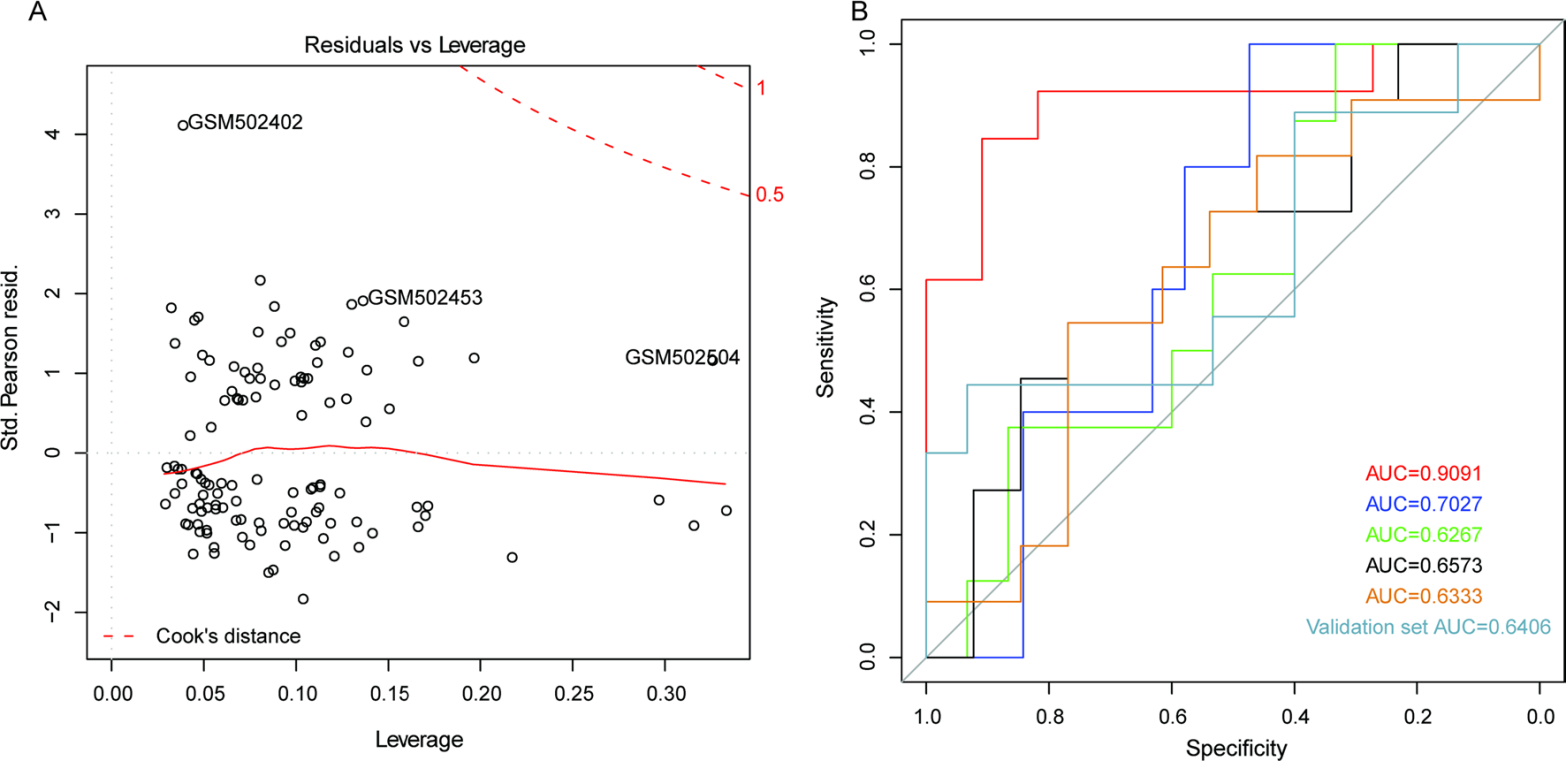
Establishment and evaluation of the logistic regression model. **A** The logistic regression model. The red dashed line indicates the COOK distance. In general, points with the COOK distance larger than 0.5 (influential points) may influence the model accuracy. **B** The ROC curve. The horizontal axis represents false positive rate (FPR), and the vertical axis represents true positive rate (TPR). AUC value could assess the performance of the model, and the high AUC value ranging from 0 to 1 indicates good performance of the model

In the first place, the logistic regression model 1 was established with the expression values of the 50 genes as variables. To construct a model with as few variables as possible, the stepwise regression analysis was conducted, which screened out 21 genes. Then the logistic regression model 2 was reconstructed with these 21 genes as variables, and it was found that there were 10 significant genes including STAT3, IL1RN, C5AR1, CXCL16, IL17RA, SLC11A1, TLR2, IL1B, LYN and CKAP4 (*p* < 0.05), indicating that these 10 genes contributed more to the model. The logistic regression model 3 was finally constructed based on the 10 genes, and there were no extreme points that could influence the accuracy of the model (Fig. 4A).

The fivefold Cross Validation method was used to evaluate the reliability of this model. The samples were randomly classified into 5 groups, and 4 groups of samples were regarded as the training sets to construct the logistic model based on the 10 genes, and another group of samples was considered as the verification set to evaluate the reliability of this model. This process was repeated for 5 times. The cross-validation process can ensure that each subsample is trained and tested, which can reduce the error and reflect the actual detection capability of the model. The results indicated that the AUCs of the 5 models in the 5 validation sets were 0.9091, 0.7027, 0.6267, 0.6573 and 0.6333, respectively, with the average AUC of 0.708 (Fig. 4B). In addition, the 16 samples analyzed by Illumina HumanHT-12 V4.0 expression bead chip were used as an validation set to determine the reliability of the model, and the results indicated that the AUC was 0.6406 (Fig. 4B). The performance of this model was also further tested in another independent validation dataset GSE43292, and the corresponding AUC value was 0.8027 (Additional file 1: Fig. S1). All these results demonstrated that the logistic regression model constructed based on these 10 genes could predict the type of samples, and might be applied to the diagnosis of patients with atherosclerosis.

## Discussion

Atherosclerosis is a significant cause of morbidity and mortality in the world [21], and investigations on the diagnostic markers are significant for the early diagnosis and high-quality treatment of atherosclerosis. A number of diagnostic makers (risk factors) in serum have been identified and studied, however, the peripheral blood samples are more readily available than the serum samples. In the present study, the peripheral blood samples were used to identify potential diagnostic makers, and 532 genes in blue module were predicted to be associated with atherosclerosis initiation based on the WGCNA analysis. Enrichment analysis revealed that genes in the blue module were primarily enriched in atherosclerosis-associated functions and pathways, including neutrophil activation, neutrophil mediated immune response and TNF signaling pathway.

The PPI network was constructed, and 50 genes which were closely associated with the occurrence of atherosclerosis were screened by MCC algorithm. In the MCC algorithm, the genes are ranked based on their importance or degree of coreness. A high score of gene indicates this gene is of great importance in the module. To construct a strong explanatory model with as few variables as possible, the stepwise regression analysis was carried out and 21 genes were screened out. Then the logistic regression model 2 was reconstructed with these 21 genes as the variables, and 10 significant genes involved in atherosclerosis including STAT3, IL1RN, C5AR1, CXCL16, IL17RA, SLC11A1, TLR2, IL1B, LYN and CKAP4 were selected. It is noted that the majority of the identified genes are reported to be associated with atherosclerosis. The signal transducer and activator of transcription 3 (STAT3), belonging to the STAT family, is conserved in structure and plays an important role in the process of atherosclerosis [22]. Abnormal activation of STAT3 regulates the progression of atherosclerosis through affecting endothelial cells, macrophages, inflammation, etc., and targeted inhibition of STAT3 is consequently considered as a potential treatment strategy for atherosclerosis. Interleukin-1 receptor antagonist (IL1RN) is a natural inhibitor of IL-1, and it has been reported that IL-1, primarily expressed in the endothelium of atherosclerotic plaques, is partly regulated by IL1RN and may be associated with the inflammatory mechanism of atherosclerosis formation [23]. Therefore, IL1RN is probably involved in atherogenesis by acting with IL-1. C5AR1 is one of the C5a receptors (C5aRs), the key pro-inflammatory mediators with various biological functions [24, 25]. The C5a-C5aR axis is demonstrated to be engaged in the development of atherosclerosis lesions through the study of humans and mice, and C5aR blockade could decrease the atherosclerotic lesion formation [25]. Chemokine CXC ligand 16 (CXCL16), a member of CXC chemokine family, has two distinct forms including the membrane-bound and soluble modalities, and is implicated in the accommodation of inflammation [26, 27]. With the characteristics including the chemoattractive, adhesive and scavenging features, CXCL16 plays a role in the atherosclerosis lesion formation, in which CXCL16 may acts as a pro-inflammatory factor [28, 29]. Interleukin-17 receptor A (IL17RA) is a receptor of IL-17, the pro-inflammatory cytokine generated by a diversity of cells and exerts its effects via binding to IL17RA [30]. Nordlohne et al. found that IL17RA was involved in the lipid metabolism and pathogenesis of atherosclerosis [31]. Butcher et al. indicated the pro-inflammatory effects of the IL17A/IL17RA axis during the progression of atherosclerosis [32]. SLC11A1 (also known as NRAMP1) is located on the phagolysosome membrane in macrophages with various functions, such as the regulatory effects on CXC chemokine, IL1B and nitric oxide release [33]. Hägg et al. have identified several novel susceptibility genes including SLC11A1 in atherosclerosis, which is differentially expressed between atherosclerosis samples and control samples through the expression profiling of macrophages [34]. TLR2, a cell surface receptor, presents obviously elevated expression level in atherosclerotic plaques, which promotes the formation of atherosclerotic plaques [35, 36]. TLR2 is involved in the early atherosclerosis in mice, and could enhance atherosclerosis via targeting the cells originate from non-bone marrow [35]. And the flow suppression for the expression of TLR2 is believed to exhibit an anti-atherosclerotic effect [37]. IL1B is an important pro-inflammatory cytokine, and is related to the progression of atherosclerosis [38]. It is implicated in atherosclerosis through inducing generation of cytokines and proteolytic enzymes, thus affecting the formation and stability of atheroma, and the anti-IL1B antibody is able to suppress the development of atherosclerosis [39]. LYN is the src family gene, and it is revealed that the scavenger receptor CD36 could bind to the oxidized LDL, thus activating LYN by the recruitment of Na/K-ATPase-LYN complex and enhancing the development of atherosclerosis [40]. Cytoskeleton associated-protein 4 (CKAP4), originally identified as an endoplasmic reticulum resident protein, is a reversibly palmitoylated, type II transmembrane protein [41]. It has been revealed that CKAP4 exerts an suppressive effect on the α5β1 integrin recycling, while the the α5β1 integrin is closely associated with atherosclerosis and plays a key role in this disease [42, 43]. Therefore, we speculate that CKAP4 may participate in atherosclerosis by regulating the the α5β1 integrin, however, deeper investigation is needed to elucidate the underlying relationships. The potential relationships between the 10 identified genes and atherosclerosis were summarized in Additional file 6: Table S5. These results confirmed our results that the 10 key genes identified in this study were closely related to the progression of atherosclerosis.

Finally, the logistic regression diagnostic model based on the 10 key genes was constructed and could predict the type of samples, which might be applied to the diagnosis of patients with atherosclerosis. Moreover, compared with other types of samples, peripheral blood samples are more easily obtained and have a certain application prospect. However, there are some limitations in this study: Firstly, due to the lack of information on the cardiac risk factors of the samples such as age and sex, the cardiac risk factors were not incorporated into the WGCNA and logistic regression analysis. This is a potential caveat of our study as the selected genes may also associate with the cardiac risk factors and not correlate to atherosclerosis directly. Secondly, although the 10 potentially key genes were identified, the specific biological functions and characteristics of these genes are needed to be further explored in the future. Thirdly, more clinical samples should be collected for further validation of our model.

## Conclusions

In summary, STAT3, IL1RN, C5AR1, CXCL16, IL17RA, SLC11A1, TLR2, IL1B, LYN and CKAP4 might be novel diagnostic signatures for atherosclerosis. The logistic regression models based on the 10 optimal key genes was constructed and could exert a better diagnostic value in atherosclerosis.

## Supplementary Information

**Additional file 1: Fig S1**. The ROC curve for GSE43292 dataset. The AUC value could assess the performance of the model, and the high AUC value ranging from 0 to 1 indicates good performance of the model.

**Additional file 2**.** Table S1**. Genes in the blue module.

**Additional file 3**.** Table S2**. Significantly enriched GO terms.

**Additional file 4**.** Table S3**. Significantly enriched KEGG pathways.

**Additional file 5**.** Table S4**. Top 50 in network string_interactions111.tsv ranked by MCC method.

**Additional file 6**.** Table S5**. The potential relationships between the 10 identified genes and atherosclerosis.

## Data Availability

The datasets generated and analysed during the current study are available in the GEO repository [https://www.ncbi.nlm.nih.gov/geo/, Accession Number: GSE20129].

## Abbreviations

CVD: Cardiovascular disease
WGCNA: Weighted gene co-expression network analysis
PPI: Protein-protein interaction
LDL: Low-density lipoprotein
LCAT: Cholesterol acyltransferase
hsCRP: High sensitivity C-reactive protein
GO: Gene ontology
KEGG: Kyoto encyclopedia of genes and genomes
MCC: Maximal clique centrality
ME: Module Eigengene
CXCL16: Chemokine CXC ligand 16
IL17RA: Interleukin-17 receptor A
STAT3: Signal transducer and activator of transcription 3
IL1RN: Interleukin-1 receptor antagonist
C5aRs: C5a receptors
IL17RA: Interleukin-17 receptor A
CKAP4: Cytoskeleton associated-protein 4
