# Supplementary figures and images for: A ten-genes-based diagnostic signature for atherosclerosis
Source: BMC Cardiovasc Disord. 2021 Oct 23;21:513. doi: 10.1186/s12872-021-02323-9 (PMC8540101; doi:10.1186/s12872-021-02323-9)

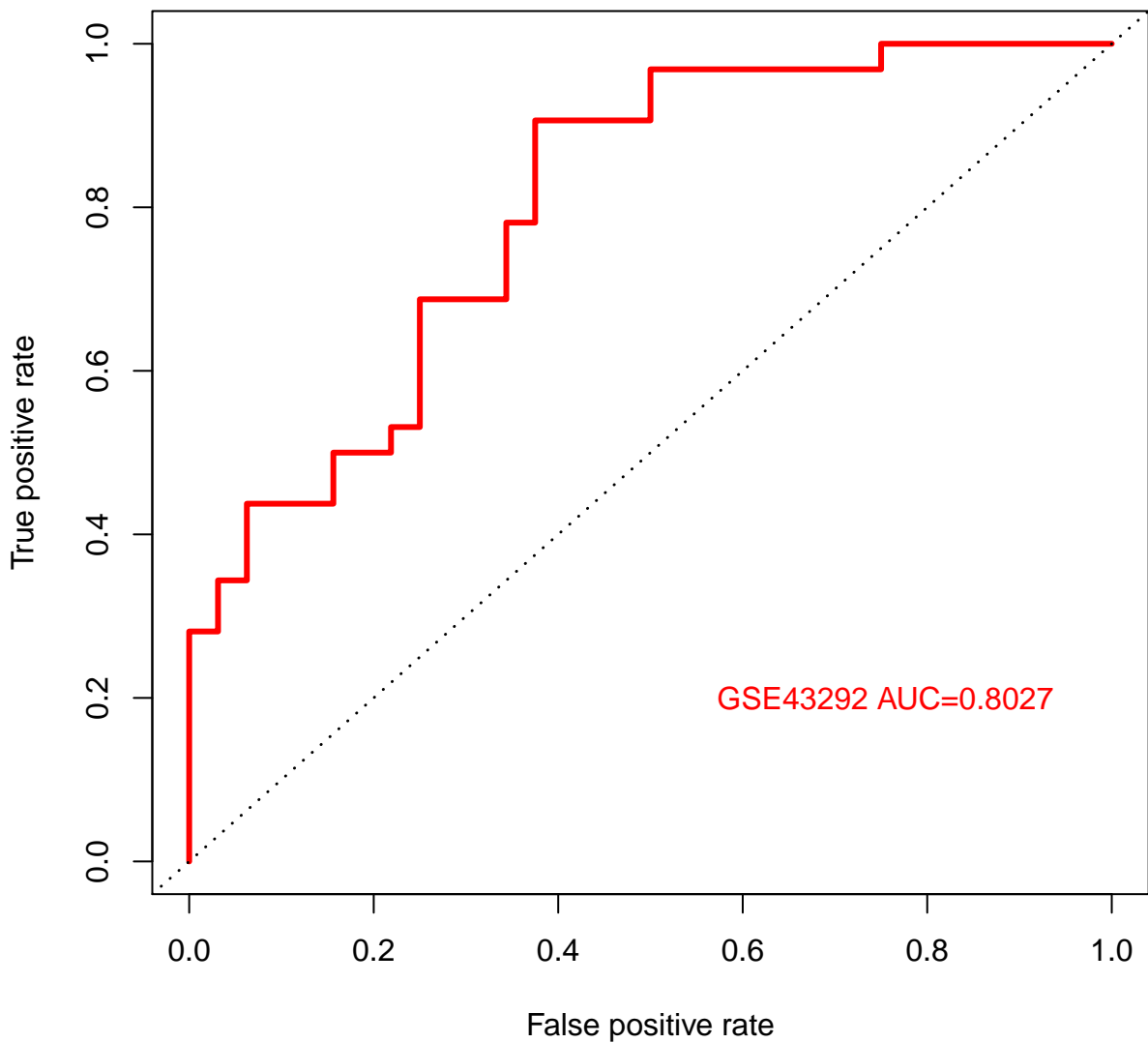

Supplement: Supplementary file 1 — Additional file 1: Fig S1. The ROC curve for GSE43292 dataset. The AUC value could assess the performance of the model, and the high AUC value ranging from 0 to 1 indicates good performance of the model. [file 12872_2021_2323_MOESM1_ESM.pdf]
