# Supplementary material for: A ten-genes-based diagnostic signature for atherosclerosis
Source: BMC Cardiovasc Disord. 2021 Oct 23;21:513. doi: 10.1186/s12872-021-02323-9 (PMC8540101; doi:10.1186/s12872-021-02323-9)
Supplement: Supplementary file 3 — Additional file 3. Table S2. Significantly enriched GO terms. [file 12872_2021_2323_MOESM3_ESM.docx]

Table S2 Significantly enriched GO terms.

| ONTOLOGY | ID | Description | GeneRatio | BgRatio | pvalue | p.adjust | qvalue | geneID | Count |
| --- | --- | --- | --- | --- | --- | --- | --- | --- | --- |
|  |  |  |  |  |  |  |  |  |  |
| BP | GO:0042119 | neutrophil activation | 58/421 | 498/17653 | 5.68E-24 | 2.38E-20 | 2.01E-20 | ARSA/APAF1/LAMP2/ARHGAP9/CANT1/FPR1/PLAUR/FCGR2A/TIMP2/F2RL1/SIRPB1/IMPDH1/RAB24/SLC15A4/ALOX5/SERPINA1/SNAP23/PECAM1/C5AR1/CD300A/PYGL/CD93/PGLYRP1/ADAM8/CDA/MGAM/LILRA2/FCGR3B/SLC11A1/SIGLEC5/QPCT/CTSS/CEACAM3/PTAFR/S100A11/SIRPA/ANPEP/MOSPD2/CR1/PADI2/TLR2/SLC2A3/PREX1/HPSE/LILRB3/CXCL1/HSPA1A/CRISPLD2/CKAP4/NFAM1/SIGLEC9/MMP9/MANBA/MVP/IQGAP1/LRG1/HSPA6/IGF2R | 58 |
| BP | GO:0002283 | neutrophil activation involved in immune response | 56/421 | 488/17653 | 7.63E-23 | 1.60E-19 | 1.35E-19 | ARSA/APAF1/LAMP2/ARHGAP9/CANT1/FPR1/PLAUR/FCGR2A/TIMP2/SIRPB1/IMPDH1/RAB24/SLC15A4/ALOX5/SERPINA1/SNAP23/PECAM1/C5AR1/CD300A/PYGL/CD93/PGLYRP1/ADAM8/CDA/MGAM/LILRA2/FCGR3B/SLC11A1/SIGLEC5/QPCT/CTSS/CEACAM3/PTAFR/S100A11/SIRPA/ANPEP/MOSPD2/CR1/PADI2/TLR2/SLC2A3/HPSE/LILRB3/CXCL1/HSPA1A/CRISPLD2/CKAP4/NFAM1/SIGLEC9/MMP9/MANBA/MVP/IQGAP1/LRG1/HSPA6/IGF2R | 56 |
| BP | GO:0002446 | neutrophil mediated immunity | 56/421 | 500/17653 | 2.51E-22 | 3.50E-19 | 2.96E-19 | ARSA/APAF1/LAMP2/ARHGAP9/CANT1/FPR1/PLAUR/FCGR2A/TIMP2/F2RL1/SIRPB1/IMPDH1/RAB24/SLC15A4/ALOX5/SERPINA1/SNAP23/PECAM1/C5AR1/CD300A/PYGL/CD93/PGLYRP1/ADAM8/CDA/MGAM/FCGR3B/SLC11A1/SIGLEC5/QPCT/CTSS/CEACAM3/PTAFR/S100A11/SIRPA/ANPEP/MOSPD2/CR1/PADI2/TLR2/SLC2A3/HPSE/LILRB3/CXCL1/HSPA1A/CRISPLD2/CKAP4/NFAM1/SIGLEC9/MMP9/MANBA/MVP/IQGAP1/LRG1/HSPA6/IGF2R | 56 |
| BP | GO:0043312 | neutrophil degranulation | 55/421 | 485/17653 | 3.38E-22 | 3.54E-19 | 3.00E-19 | ARSA/APAF1/LAMP2/ARHGAP9/CANT1/FPR1/PLAUR/FCGR2A/TIMP2/SIRPB1/IMPDH1/RAB24/SLC15A4/ALOX5/SERPINA1/SNAP23/PECAM1/C5AR1/CD300A/PYGL/CD93/PGLYRP1/ADAM8/CDA/MGAM/FCGR3B/SLC11A1/SIGLEC5/QPCT/CTSS/CEACAM3/PTAFR/S100A11/SIRPA/ANPEP/MOSPD2/CR1/PADI2/TLR2/SLC2A3/HPSE/LILRB3/CXCL1/HSPA1A/CRISPLD2/CKAP4/NFAM1/SIGLEC9/MMP9/MANBA/MVP/IQGAP1/LRG1/HSPA6/IGF2R | 55 |
| BP | GO:0002237 | response to molecule of bacterial origin | 32/421 | 345/17653 | 5.70E-11 | 4.77E-08 | 4.04E-08 | FOS/TNFRSF10C/PTGS2/PELI1/TNFRSF1A/UPF1/CDK4/C5AR1/CEBPB/ALPL/TLR4/ADM/LITAF/LILRA2/TLR5/NOTCH1/TLR1/SLC11A1/PTAFR/TLR6/IFNAR1/THBD/TRIB1/TLR2/IL1B/LYN/PDE4B/CXCL1/HCK/CSF2RB/SBNO2/PLCG2 | 32 |
| BP | GO:0032496 | response to lipopolysaccharide | 30/421 | 327/17653 | 3.02E-10 | 2.11E-07 | 1.79E-07 | FOS/TNFRSF10C/PTGS2/PELI1/TNFRSF1A/UPF1/CDK4/C5AR1/CEBPB/ALPL/TLR4/ADM/LITAF/LILRA2/TLR5/NOTCH1/SLC11A1/PTAFR/IFNAR1/THBD/TRIB1/TLR2/IL1B/LYN/PDE4B/CXCL1/HCK/CSF2RB/SBNO2/PLCG2 | 30 |
| BP | GO:0001819 | positive regulation of cytokine production | 33/421 | 411/17653 | 1.22E-09 | 7.30E-07 | 6.18E-07 | TLR8/FLOT1/BCL3/SULF2/F2RL1/PTGS2/PELI1/IL1RAP/C5AR1/CEBPB/IL6R/RNF135/TLR4/ADAM8/NLRP12/TLR5/IL17RA/TLR1/SEC14L1/SLC11A1/PTAFR/TLR6/FFAR2/PRKDC/CLEC4E/TLR2/IL1B/STAT5B/HPSE/PDE4B/HSPA1A/NFAM1/PLCG2 | 33 |
| BP | GO:0045088 | regulation of innate immune response | 33/421 | 427/17653 | 3.19E-09 | 1.67E-06 | 1.41E-06 | RPS6KA5/TLR8/FLOT1/RNASEL/F2RL1/PELI1/CD300LF/CD300A/RNF135/PGLYRP1/TLR4/ADAM8/LILRA2/ICAM3/TLR5/TLR1/SEC14L1/CTSS/TLR6/IFNAR1/FFAR2/IFNGR2/CR1/PAK1/PRKDC/CLEC4E/TLR2/STAT5B/LYN/HSPA1A/HCK/RAF1/PLCG2 | 33 |
| BP | GO:0031349 | positive regulation of defense response | 34/421 | 466/17653 | 7.87E-09 | 3.66E-06 | 3.10E-06 | RPS6KA5/TLR8/FLOT1/F2RL1/PTGS2/PELI1/CD300LF/TNFRSF1A/CD300A/PGLYRP1/TLR4/ADAM8/NLRP12/LILRA2/ICAM3/TLR5/IL17RA/TLR1/SEC14L1/CTSS/TLR6/FFAR2/PAK1/PRKDC/CLEC4E/TLR2/IL1B/STAT5B/OSM/LYN/HSPA1A/HCK/RAF1/PLCG2 | 34 |
| BP | GO:0034121 | regulation of toll-like receptor signaling pathway | 12/421 | 60/17653 | 1.44E-08 | 6.03E-06 | 5.11E-06 | FLOT1/F2RL1/PELI1/CD300LF/CD300A/TLR4/LILRA2/TLR5/TLR1/TLR6/TLR2/LYN | 12 |
| BP | GO:0002218 | activation of innate immune response | 26/421 | 301/17653 | 1.67E-08 | 6.35E-06 | 5.38E-06 | RPS6KA5/TLR8/FLOT1/F2RL1/PELI1/CD300LF/CD300A/PGLYRP1/TLR4/LILRA2/ICAM3/TLR5/TLR1/SEC14L1/CTSS/TLR6/FFAR2/PAK1/PRKDC/CLEC4E/TLR2/LYN/HSPA1A/HCK/RAF1/PLCG2 | 26 |
| BP | GO:0002758 | innate immune response-activating signal transduction | 25/421 | 283/17653 | 2.02E-08 | 7.06E-06 | 5.98E-06 | RPS6KA5/TLR8/FLOT1/F2RL1/PELI1/CD300LF/CD300A/PGLYRP1/TLR4/LILRA2/ICAM3/TLR5/TLR1/SEC14L1/CTSS/TLR6/FFAR2/PAK1/CLEC4E/TLR2/LYN/HSPA1A/HCK/RAF1/PLCG2 | 25 |
| BP | GO:0045408 | regulation of interleukin-6 biosynthetic process | 7/421 | 15/17653 | 2.28E-08 | 7.34E-06 | 6.22E-06 | CEBPB/GHRL/NLRP12/TLR1/PTAFR/TLR6/IL1B | 7 |
| BP | GO:0045089 | positive regulation of innate immune response | 28/421 | 353/17653 | 2.96E-08 | 8.87E-06 | 7.51E-06 | RPS6KA5/TLR8/FLOT1/F2RL1/PELI1/CD300LF/CD300A/PGLYRP1/TLR4/ADAM8/LILRA2/ICAM3/TLR5/TLR1/SEC14L1/CTSS/TLR6/FFAR2/PAK1/PRKDC/CLEC4E/TLR2/STAT5B/LYN/HSPA1A/HCK/RAF1/PLCG2 | 28 |
| BP | GO:0050900 | leukocyte migration | 33/421 | 470/17653 | 3.33E-08 | 9.29E-06 | 7.87E-06 | TNFSF14/FPR1/F2RL1/TREM1/PECAM1/C5AR1/CD300A/IL6R/ADAM8/NLRP12/CXCL16/IL17RA/CEACAM3/PTAFR/FFAR2/SIRPA/THBD/MOSPD2/PIK3CD/PADI2/IL1B/PREX1/STAT5B/LYN/PDE4B/NOV/CXCL1/CSF3R/HCK/ITGA5/MMP9/F11R/DAPK2 | 33 |
| BP | GO:0042226 | interleukin-6 biosynthetic process | 7/421 | 17/17653 | 6.61E-08 | 1.73E-05 | 1.46E-05 | CEBPB/GHRL/NLRP12/TLR1/PTAFR/TLR6/IL1B | 7 |
| BP | GO:0032637 | interleukin-8 production | 12/421 | 70/17653 | 8.83E-08 | 2.17E-05 | 1.84E-05 | TLR8/BCL3/F2RL1/TLR4/LILRA2/TLR5/TLR1/TLR6/FFAR2/TLR2/IL1B/HSPA1A | 12 |
| BP | GO:0032103 | positive regulation of response to external stimulus | 24/421 | 289/17653 | 1.25E-07 | 2.92E-05 | 2.47E-05 | TNFSF14/STX3/F2RL1/PTGS2/TNFRSF1A/C5AR1/GHRL/IL6R/NDEL1/TLR4/ADAM8/NLRP12/IL17RA/TLR6/PPM1F/FFAR2/THBD/MOSPD2/TLR2/IL1B/STAT5B/OSM/CXCL1/DAPK2 | 24 |
| BP | GO:0032677 | regulation of interleukin-8 production | 11/421 | 63/17653 | 2.52E-07 | 5.55E-05 | 4.70E-05 | TLR8/BCL3/F2RL1/TLR4/TLR5/TLR1/TLR6/FFAR2/TLR2/IL1B/HSPA1A | 11 |
| BP | GO:0071260 | cellular response to mechanical stimulus | 12/421 | 78/17653 | 3.03E-07 | 6.35E-05 | 5.38E-05 | TNFSF14/TLR8/PTGS2/TNFRSF1A/TLR4/TLR5/MAP2K4/HDAC4/MAP3K2/IL1B/KCNJ2/SLC9A1 | 12 |
| BP | GO:0002221 | pattern recognition receptor signaling pathway | 18/421 | 181/17653 | 3.49E-07 | 6.96E-05 | 5.90E-05 | TLR8/FLOT1/F2RL1/PELI1/CD300LF/CD300A/PGLYRP1/TLR4/LILRA2/TLR5/TLR1/SEC14L1/CTSS/TLR6/FFAR2/TLR2/LYN/HSPA1A | 18 |
| BP | GO:0071496 | cellular response to external stimulus | 24/421 | 307/17653 | 3.83E-07 | 7.18E-05 | 6.08E-05 | TNFSF14/TLR8/LAMP2/NCOA1/FOS/RALB/PTGS2/TNFRSF1A/TLR4/TLR5/MAP3K5/ULK1/FNIP1/MAP2K4/HDAC4/SH3GLB1/MAP3K2/IL1B/KCNJ2/LYN/DSC2/NUAK2/SLC9A1/CBS | 24 |
| BP | GO:0030595 | leukocyte chemotaxis | 19/421 | 202/17653 | 3.94E-07 | 7.18E-05 | 6.08E-05 | TNFSF14/F2RL1/C5AR1/IL6R/ADAM8/CXCL16/IL17RA/FFAR2/MOSPD2/PIK3CD/PADI2/IL1B/PREX1/LYN/PDE4B/NOV/CXCL1/CSF3R/DAPK2 | 19 |
| BP | GO:0071216 | cellular response to biotic stimulus | 19/421 | 204/17653 | 4.59E-07 | 8.01E-05 | 6.79E-05 | APAF1/UPF1/CDK4/CEBPB/TLR4/LITAF/LILRA2/TLR5/TLR1/PTAFR/TLR6/TRIB1/TLR2/IL1B/GSK3B/LYN/PDE4B/HCK/SBNO2 | 19 |
| BP | GO:0060326 | cell chemotaxis | 22/421 | 273/17653 | 7.04E-07 | 0.000118 | 1.00E-04 | TNFSF14/FPR1/F2RL1/C5AR1/EPHB1/IL6R/ADAM8/CXCL16/IL17RA/NOTCH1/FFAR2/MOSPD2/PIK3CD/PADI2/IL1B/PREX1/LYN/PDE4B/NOV/CXCL1/CSF3R/DAPK2 | 22 |
| BP | GO:0032757 | positive regulation of interleukin-8 production | 9/421 | 45/17653 | 9.49E-07 | 0.000153 | 0.000129 | TLR8/F2RL1/TLR4/TLR5/TLR1/FFAR2/TLR2/IL1B/HSPA1A | 9 |
| BP | GO:0032635 | interleukin-6 production | 14/421 | 120/17653 | 1.04E-06 | 0.000162 | 0.000137 | TLR8/F2RL1/IL1RAP/CEBPB/GHRL/IL6R/TLR4/NLRP12/LILRA2/TLR1/PTAFR/TLR6/TLR2/IL1B | 14 |
| BP | GO:0097529 | myeloid leukocyte migration | 17/421 | 177/17653 | 1.21E-06 | 0.00018 | 0.000153 | PECAM1/C5AR1/CD300A/IL6R/ADAM8/IL17RA/MOSPD2/PIK3CD/IL1B/PREX1/STAT5B/LYN/PDE4B/NOV/CXCL1/CSF3R/DAPK2 | 17 |
| BP | GO:0002755 | MyD88-dependent toll-like receptor signaling pathway | 8/421 | 36/17653 | 1.65E-06 | 0.000238 | 0.000202 | TLR8/CD300LF/CD300A/TLR4/TLR5/TLR1/TLR6/TLR2 | 8 |
| BP | GO:0071219 | cellular response to molecule of bacterial origin | 17/421 | 182/17653 | 1.78E-06 | 0.000248 | 0.00021 | UPF1/CDK4/CEBPB/TLR4/LITAF/LILRA2/TLR5/TLR1/PTAFR/TLR6/TRIB1/TLR2/IL1B/LYN/PDE4B/HCK/SBNO2 | 17 |
| BP | GO:0002521 | leukocyte differentiation | 30/421 | 483/17653 | 1.88E-06 | 0.000255 | 0.000216 | BCL3/STAT3/GAB2/FOS/F2RL1/TFRC/RASSF2/CEBPB/PGLYRP1/TLR4/ADAM8/LGALS1/BCL6/FNIP1/HDAC4/PIK3CD/CR1/TRIB1/PRKDC/CLEC4E/TLR2/PREX1/STAT5B/LYN/LILRB3/NFAM1/MMP9/CDK6/SBNO2/PLCG2 | 30 |
| BP | GO:1901216 | positive regulation of neuron death | 11/421 | 78/17653 | 2.31E-06 | 0.000302 | 0.000256 | FOS/PICALM/TLR4/ZNF746/MAP3K5/CDK5R1/MAP2K4/TLR6/HDAC4/PIN1/GSK3B | 11 |
| BP | GO:0032675 | regulation of interleukin-6 production | 13/421 | 111/17653 | 2.42E-06 | 0.000308 | 0.000261 | TLR8/F2RL1/IL1RAP/CEBPB/GHRL/IL6R/TLR4/NLRP12/TLR1/PTAFR/TLR6/TLR2/IL1B | 13 |
| BP | GO:0002224 | toll-like receptor signaling pathway | 14/421 | 135/17653 | 4.29E-06 | 0.000529 | 0.000448 | TLR8/FLOT1/F2RL1/PELI1/CD300LF/CD300A/TLR4/LILRA2/TLR5/TLR1/CTSS/TLR6/TLR2/LYN | 14 |
| BP | GO:0034123 | positive regulation of toll-like receptor signaling pathway | 6/421 | 20/17653 | 5.18E-06 | 0.000617 | 0.000523 | FLOT1/F2RL1/PELI1/TLR5/TLR1/TLR2 | 6 |
| BP | GO:0097530 | granulocyte migration | 13/421 | 119/17653 | 5.30E-06 | 0.000617 | 0.000523 | PECAM1/C5AR1/CD300A/ADAM8/IL17RA/MOSPD2/PIK3CD/IL1B/PREX1/PDE4B/CXCL1/CSF3R/DAPK2 | 13 |
| BP | GO:0042089 | cytokine biosynthetic process | 12/421 | 105/17653 | 7.63E-06 | 0.000864 | 0.000732 | TLR8/BCL3/IL1RAP/CEBPB/GHRL/TLR4/NLRP12/TLR1/PTAFR/TLR6/IL1B/STAT5B | 12 |
| BP | GO:0034134 | toll-like receptor 2 signaling pathway | 5/421 | 13/17653 | 8.28E-06 | 0.000905 | 0.000767 | F2RL1/TLR1/TLR6/TLR2/LYN | 5 |
| BP | GO:0042107 | cytokine metabolic process | 12/421 | 106/17653 | 8.43E-06 | 0.000905 | 0.000767 | TLR8/BCL3/IL1RAP/CEBPB/GHRL/TLR4/NLRP12/TLR1/PTAFR/TLR6/IL1B/STAT5B | 12 |
| BP | GO:0032755 | positive regulation of interleukin-6 production | 10/421 | 74/17653 | 9.84E-06 | 0.001031 | 0.000873 | TLR8/F2RL1/IL1RAP/IL6R/TLR4/TLR1/PTAFR/TLR6/TLR2/IL1B | 10 |
| BP | GO:0098543 | detection of other organism | 5/421 | 14/17653 | 1.26E-05 | 0.00129 | 0.001093 | PGLYRP1/TLR4/TLR1/TLR6/TLR2 | 5 |
| BP | GO:0042035 | regulation of cytokine biosynthetic process | 11/421 | 95/17653 | 1.61E-05 | 0.001603 | 0.001358 | TLR8/BCL3/CEBPB/GHRL/TLR4/NLRP12/TLR1/PTAFR/TLR6/IL1B/STAT5B | 11 |
| BP | GO:0050707 | regulation of cytokine secretion | 15/421 | 172/17653 | 1.65E-05 | 0.001603 | 0.001358 | TLR8/F2RL1/IL1RAP/GHRL/TLR4/NLRP12/TLR5/IL17RA/TLR1/TLR6/FFAR2/CLEC4E/TLR2/IL1B/LYN | 15 |
| BP | GO:0044262 | cellular carbohydrate metabolic process | 19/421 | 262/17653 | 1.83E-05 | 0.001741 | 0.001475 | PFKFB4/STAT3/DGAT2/PYGL/MGAM/HK2/GK/IMPA2/INPP5A/B3GNT8/MIDN/PTAFR/IRS2/HDAC4/PDK3/GSK3B/PGD/MANBA/NDST1 | 19 |
| BP | GO:0071222 | cellular response to lipopolysaccharide | 15/421 | 174/17653 | 1.89E-05 | 0.001745 | 0.001478 | UPF1/CDK4/CEBPB/TLR4/LITAF/LILRA2/TLR5/PTAFR/TRIB1/TLR2/IL1B/LYN/PDE4B/HCK/SBNO2 | 15 |
| BP | GO:0034122 | negative regulation of toll-like receptor signaling pathway | 7/421 | 36/17653 | 1.92E-05 | 0.001745 | 0.001478 | F2RL1/CD300LF/CD300A/TLR4/LILRA2/TLR6/LYN | 7 |
| BP | GO:0050663 | cytokine secretion | 16/421 | 196/17653 | 1.98E-05 | 0.001765 | 0.001495 | TLR8/F2RL1/IL1RAP/GHRL/TLR4/NLRP12/TLR5/IL17RA/NOTCH1/TLR1/TLR6/FFAR2/CLEC4E/TLR2/IL1B/LYN | 16 |
| BP | GO:1990266 | neutrophil migration | 11/421 | 99/17653 | 2.39E-05 | 0.002084 | 0.001765 | PECAM1/C5AR1/ADAM8/MOSPD2/PIK3CD/IL1B/PREX1/PDE4B/CXCL1/CSF3R/DAPK2 | 11 |
| BP | GO:0045785 | positive regulation of cell adhesion | 24/421 | 392/17653 | 2.57E-05 | 0.002201 | 0.001864 | TNFSF14/STX3/FLOT1/TFRC/SIRPB1/ADAM8/FLCN/LGALS1/FLOT2/BCL6/PTAFR/DOCK5/PPM1F/SIRPA/PAK1/IL1B/PREX1/STAT5B/GSK3B/LYN/ITGA5/CDK6/IQGAP1/NINJ1 | 24 |
| BP | GO:0031663 | lipopolysaccharide-mediated signaling pathway | 8/421 | 54/17653 | 3.90E-05 | 0.003265 | 0.002766 | TLR4/LILRA2/PTAFR/TRIB1/TLR2/IL1B/LYN/HCK | 8 |
| BP | GO:0050729 | positive regulation of inflammatory response | 12/421 | 124/17653 | 4.15E-05 | 0.003406 | 0.002886 | PTGS2/TNFRSF1A/TLR4/ADAM8/NLRP12/IL17RA/TLR6/FFAR2/TLR2/IL1B/STAT5B/OSM | 12 |
| BP | GO:0033002 | muscle cell proliferation | 16/421 | 211/17653 | 4.88E-05 | 0.003928 | 0.003327 | STAT3/PTGS2/RXRA/EPHB1/IL6R/MAP3K5/NOTCH1/PTAFR/JARID2/HDAC4/PAK1/TRIB1/PRKDC/NOV/CFLAR/MMP9 | 16 |
| BP | GO:0002573 | myeloid leukocyte differentiation | 15/421 | 191/17653 | 5.60E-05 | 0.004429 | 0.003752 | GAB2/FOS/F2RL1/TFRC/RASSF2/CEBPB/TLR4/PIK3CD/TRIB1/TLR2/LYN/LILRB3/MMP9/CDK6/SBNO2 | 15 |
| BP | GO:0042108 | positive regulation of cytokine biosynthetic process | 8/421 | 57/17653 | 5.81E-05 | 0.004511 | 0.003821 | TLR8/BCL3/TLR4/TLR1/PTAFR/TLR6/IL1B/STAT5B | 8 |
| BP | GO:0002328 | pro-B cell differentiation | 4/421 | 10/17653 | 5.98E-05 | 0.004551 | 0.003855 | FLCN/NOTCH1/FNIP1/PRKDC | 4 |
| BP | GO:0050764 | regulation of phagocytosis | 9/421 | 74/17653 | 6.43E-05 | 0.004808 | 0.004073 | F2RL1/SIRPB1/CD300LF/CD300A/SLC11A1/SIRPA/TLR2/IL1B/HCK | 9 |
| BP | GO:0098581 | detection of external biotic stimulus | 5/421 | 19/17653 | 6.65E-05 | 0.004884 | 0.004137 | PGLYRP1/TLR4/TLR1/TLR6/TLR2 | 5 |
| BP | GO:0042326 | negative regulation of phosphorylation | 26/421 | 473/17653 | 7.37E-05 | 0.005326 | 0.004511 | STAT3/SH3BP5L/RNF149/F2RL1/RASSF2/CDK4/CD300A/TLR4/NLRP12/FLCN/STRAP/MIDN/PPM1F/SORL1/IRS2/HDAC4/TRIB1/PRKDC/IL1B/PIN1/PREX1/LYN/DUSP1/CDK6/MVP/FAM129A | 26 |
| BP | GO:0070997 | neuron death | 20/421 | 316/17653 | 7.67E-05 | 0.005444 | 0.004611 | APAF1/STAT3/FOS/PICALM/C5AR1/EPHB1/CEBPB/TLR4/ZNF746/ADAM8/MAP3K5/CDK5R1/MAP2K4/TLR6/EGLN1/SORL1/HDAC4/PIN1/GSK3B/SLC9A1 | 20 |
| BP | GO:0043405 | regulation of MAP kinase activity | 21/421 | 344/17653 | 8.54E-05 | 0.005878 | 0.004979 | FPR1/C5AR1/MAP3K3/CD300A/GHRL/TLR4/ADAM8/MAP3K5/MAP2K4/TLR6/SORL1/MAP3K2/PAK1/TRIB1/PROK2/IL1B/LYN/DUSP1/IQGAP1/TGFA/RAF1 | 21 |
| BP | GO:0003012 | muscle system process | 25/421 | 450/17653 | 8.56E-05 | 0.005878 | 0.004979 | SULF2/PTGS2/COL4A3BP/PXN/GHRL/MYBPH/NDUFS6/TPCN2/MAP2K4/PTAFR/DOCK5/JARID2/HDAC4/PAK1/MYBPC3/PROK2/IL1B/PIN1/KCNJ2/DSC2/PDE4B/CFLAR/SLC9A1/CAMK2G/DYSF | 25 |
| BP | GO:0045730 | respiratory burst | 6/421 | 32/17653 | 9.51E-05 | 0.006423 | 0.005441 | NCF1/SLC11A1/NCF4/PIK3CD/HCK/NCF2 | 6 |
| BP | GO:0050715 | positive regulation of cytokine secretion | 11/421 | 116/17653 | 0.000104 | 0.006802 | 0.005762 | TLR8/F2RL1/IL1RAP/TLR4/NLRP12/IL17RA/TLR1/FFAR2/CLEC4E/TLR2/IL1B | 11 |
| BP | GO:0042113 | B cell activation | 18/421 | 273/17653 | 0.000104 | 0.006802 | 0.005762 | SKAP2/BCL3/TFRC/PELI1/LAT2/CD300A/TLR4/LGALS1/BCL6/FNIP1/IRS2/HDAC4/PIK3CD/PRKDC/STAT5B/LYN/NFAM1/PLCG2 | 18 |
| BP | GO:0030335 | positive regulation of cell migration | 26/421 | 486/17653 | 0.000114 | 0.007374 | 0.006247 | TNFSF14/F2RL1/PTGS2/C5AR1/MAP3K3/IL6R/NUMB/ADAM8/CXCL16/NOTCH1/PTAFR/DOCK5/PPM1F/IRS2/HDAC4/MOSPD2/PIK3CD/PAK1/IL1B/PREX1/LYN/CXCL1/ITGA5/MMP9/DAPK2/IQGAP1 | 26 |
| BP | GO:1901653 | cellular response to peptide | 21/421 | 353/17653 | 0.000123 | 0.007784 | 0.006594 | STAT3/RNASEL/ADCY4/PXN/CDK4/GHRL/NDEL1/TLR4/ATP6V0B/TLR6/IRS2/PAK1/PRKDC/IL1B/STAT5B/GSK3B/LYN/ATP6V1B2/CFLAR/ATP6V0D1/SLC9A1 | 21 |
| BP | GO:0006953 | acute-phase response | 7/421 | 48/17653 | 0.000132 | 0.008008 | 0.006783 | STAT3/PTGS2/SERPINA1/IL1RN/CEBPB/IL6R/IL1B | 7 |
| BP | GO:0042110 | T cell activation | 25/421 | 463/17653 | 0.000134 | 0.008008 | 0.006783 | TNFSF14/BCL3/STAT3/F2RL1/TFRC/SIRPB1/PELI1/CEBPB/CD300A/ADAM8/LGALS1/FLOT2/BCL6/SLC11A1/TREML2/SIRPA/PIK3CD/CR1/PAK1/PRKDC/CLEC4E/IL1B/PREX1/STAT5B/LYN | 25 |
| BP | GO:1901652 | response to peptide | 26/421 | 491/17653 | 0.000135 | 0.008008 | 0.006783 | STAT3/RNASEL/PTGS2/ADCY4/PXN/CDK4/GHRL/NDEL1/TLR4/ADM/ATP6V0B/NOTCH1/TLR6/IRS2/PAK1/PRKDC/TLR2/IL1B/STAT5B/GSK3B/LYN/ATP6V1B2/CFLAR/ATP6V0D1/SLC9A1/IQGAP1 | 26 |
| BP | GO:0032490 | detection of molecule of bacterial origin | 4/421 | 12/17653 | 0.000136 | 0.008008 | 0.006783 | TLR4/TLR1/TLR6/TLR2 | 4 |
| BP | GO:0018212 | peptidyl-tyrosine modification | 22/421 | 382/17653 | 0.000136 | 0.008008 | 0.006783 | TPST1/STAT3/UNC119/SH3BP5L/SLA/TNFRSF1A/EPHB1/CD300A/GHRL/IL6R/NCF1/MAP2K4/CSF2RA/STAT5B/OSM/LYN/HCK/ITGA5/CSF2RB/MVP/IQGAP1/TGFA | 22 |
| BP | GO:0006909 | phagocytosis | 20/421 | 330/17653 | 0.000138 | 0.008037 | 0.006808 | FCGR2A/RAB20/F2RL1/SIRPB1/CD300LF/PECAM1/CD300A/CD93/CD302/ICAM3/SLC11A1/NCF4/SIRPA/PAK1/TLR2/IL1B/LYN/HCK/NCF2/PLCG2 | 20 |
| BP | GO:0009595 | detection of biotic stimulus | 5/421 | 22/17653 | 0.000142 | 0.008141 | 0.006896 | PGLYRP1/TLR4/TLR1/TLR6/TLR2 | 5 |
| BP | GO:0007249 | I-kappaB kinase/NF-kappaB signaling | 17/421 | 256/17653 | 0.000148 | 0.008292 | 0.007024 | TLR8/BCL3/CANT1/F2RL1/PELI1/TNFRSF1A/MAP3K3/TLR4/LITAF/NLRP12/USP10/LGALS1/TLR6/TLR2/IL1B/PELI2/CFLAR | 17 |
| BP | GO:1901214 | regulation of neuron death | 18/421 | 281/17653 | 0.000149 | 0.008292 | 0.007024 | STAT3/FOS/PICALM/C5AR1/EPHB1/CEBPB/TLR4/ZNF746/ADAM8/MAP3K5/CDK5R1/MAP2K4/TLR6/EGLN1/SORL1/HDAC4/PIN1/GSK3B | 18 |
| BP | GO:0042116 | macrophage activation | 8/421 | 65/17653 | 0.00015 | 0.008292 | 0.007024 | TLR8/CD93/TLR4/TLR1/SLC11A1/TLR6/TLR2/SBNO2 | 8 |
| BP | GO:0052548 | regulation of endopeptidase activity | 23/421 | 414/17653 | 0.000163 | 0.008889 | 0.00753 | TNFSF14/APAF1/STAT3/PLAUR/TIMP2/TNFRSF10C/PTGS2/PICALM/SERPINA1/PI3/RPS6KA1/NLRP12/MAP3K5/FNIP1/PPM1F/SORL1/CR1/LYN/CFLAR/DNAJB6/MMP9/MEFV/RAF1 | 23 |
| BP | GO:0046777 | protein autophosphorylation | 16/421 | 235/17653 | 0.000173 | 0.009281 | 0.007862 | SLA/RASSF2/MAP3K3/EPHB1/PIM3/NLRP12/ULK1/PAK1/GSK3B/LYN/HCK/MVP/DAPK2/IQGAP1/MAK/CAMK2G | 16 |
| BP | GO:0060396 | growth hormone receptor signaling pathway | 5/421 | 23/17653 | 0.000178 | 0.009425 | 0.007984 | STAT3/PXN/GHRL/STAT5B/LYN | 5 |
| BP | GO:0032611 | interleukin-1 beta production | 8/421 | 67/17653 | 0.000187 | 0.009766 | 0.008273 | TLR8/F2RL1/GHRL/TLR4/NLRP12/TLR6/IL1B/MEFV | 8 |
| BP | GO:0016045 | detection of bacterium | 4/421 | 13/17653 | 0.000192 | 0.00994 | 0.00842 | PGLYRP1/TLR1/TLR6/TLR2 | 4 |
| BP | GO:0071621 | granulocyte chemotaxis | 10/421 | 105/17653 | 0.000205 | 0.010483 | 0.00888 | C5AR1/IL17RA/MOSPD2/PIK3CD/IL1B/PREX1/PDE4B/CXCL1/CSF3R/DAPK2 | 10 |
| BP | GO:0030099 | myeloid cell differentiation | 22/421 | 394/17653 | 0.00021 | 0.010613 | 0.00899 | STAT3/GAB2/FOS/F2RL1/TFRC/RASSF2/CEBPB/TLR4/HIST1H3D/BCL6/PIK3CD/TRIB1/TLR2/STAT5B/LYN/LILRB3/CSF3R/HSPA1A/MMP9/ACTN1/CDK6/SBNO2 | 22 |
| BP | GO:0071378 | cellular response to growth hormone stimulus | 5/421 | 24/17653 | 0.00022 | 0.01085 | 0.009191 | STAT3/PXN/GHRL/STAT5B/LYN | 5 |
| BP | GO:2000482 | regulation of interleukin-8 secretion | 5/421 | 24/17653 | 0.00022 | 0.01085 | 0.009191 | F2RL1/TLR1/TLR6/FFAR2/TLR2 | 5 |
| BP | GO:0090257 | regulation of muscle system process | 16/421 | 242/17653 | 0.000241 | 0.011754 | 0.009956 | PTGS2/GHRL/MYBPH/PTAFR/DOCK5/JARID2/HDAC4/PAK1/MYBPC3/PROK2/PIN1/KCNJ2/DSC2/PDE4B/SLC9A1/CAMK2G | 16 |
| BP | GO:0030593 | neutrophil chemotaxis | 9/421 | 88/17653 | 0.000248 | 0.011934 | 0.010109 | C5AR1/MOSPD2/PIK3CD/IL1B/PREX1/PDE4B/CXCL1/CSF3R/DAPK2 | 9 |
| BP | GO:0006022 | aminoglycan metabolic process | 13/421 | 172/17653 | 0.000253 | 0.012056 | 0.010212 | ABCC5/B4GALT5/CTBS/PGLYRP1/ST3GAL2/B3GNT8/ST3GAL4/IDS/IL1B/HPSE/CHSY1/SLC9A1/NDST1 | 13 |
| BP | GO:0070304 | positive regulation of stress-activated protein kinase signaling cascade | 12/421 | 152/17653 | 0.000291 | 0.013713 | 0.011616 | F2RL1/RASSF2/MAP3K3/NCF1/TLR4/MAP3K5/MAP2K4/TLR6/MAP3K2/PAK1/IL1B/LYN | 12 |
| BP | GO:0002685 | regulation of leukocyte migration | 13/421 | 176/17653 | 0.000317 | 0.014553 | 0.012327 | TNFSF14/F2RL1/C5AR1/CD300A/IL6R/ADAM8/PTAFR/MOSPD2/PADI2/LYN/NOV/CXCL1/DAPK2 | 13 |
| BP | GO:0043154 | negative regulation of cysteine-type endopeptidase activity involved in apoptotic process | 9/421 | 91/17653 | 0.000319 | 0.014553 | 0.012327 | TNFSF14/PLAUR/TNFRSF10C/PTGS2/RPS6KA1/FNIP1/DNAJB6/MMP9/RAF1 | 9 |
| BP | GO:0018108 | peptidyl-tyrosine phosphorylation | 21/421 | 379/17653 | 0.000323 | 0.014553 | 0.012327 | STAT3/UNC119/SH3BP5L/SLA/TNFRSF1A/EPHB1/CD300A/GHRL/IL6R/NCF1/MAP2K4/CSF2RA/STAT5B/OSM/LYN/HCK/ITGA5/CSF2RB/MVP/IQGAP1/TGFA | 21 |
| BP | GO:0002688 | regulation of leukocyte chemotaxis | 10/421 | 111/17653 | 0.000323 | 0.014553 | 0.012327 | TNFSF14/F2RL1/C5AR1/IL6R/MOSPD2/PADI2/LYN/NOV/CXCL1/DAPK2 | 10 |
| BP | GO:2000484 | positive regulation of interleukin-8 secretion | 4/421 | 15/17653 | 0.000353 | 0.015743 | 0.013336 | F2RL1/TLR1/FFAR2/TLR2 | 4 |
| BP | GO:0050727 | regulation of inflammatory response | 22/421 | 411/17653 | 0.000377 | 0.016623 | 0.014081 | PTGS2/TNFRSF1A/C5AR1/GHRL/PGLYRP1/TLR4/ADAM8/NLRP12/IL17RA/BCL6/TLR6/FFAR2/CR1/TLR2/IL1B/STAT5B/OSM/LYN/NOV/HCK/MEFV/SBNO2 | 22 |
| BP | GO:0010575 | positive regulation of vascular endothelial growth factor production | 5/421 | 27/17653 | 0.000394 | 0.016869 | 0.014289 | SULF2/PTGS2/C5AR1/IL1B/HPSE | 5 |
| BP | GO:0002532 | production of molecular mediator involved in inflammatory response | 7/421 | 57/17653 | 0.000395 | 0.016869 | 0.014289 | ALOX5/SNAP23/TLR4/IL17RA/TLR6/LYN/MEFV | 7 |
| BP | GO:0045576 | mast cell activation | 7/421 | 57/17653 | 0.000395 | 0.016869 | 0.014289 | GAB2/CD300LF/SNAP23/LAT2/CD300A/PIK3CD/LYN | 7 |
| BP | GO:0052547 | regulation of peptidase activity | 23/421 | 441/17653 | 0.000403 | 0.017047 | 0.01444 | TNFSF14/APAF1/STAT3/PLAUR/TIMP2/TNFRSF10C/PTGS2/PICALM/SERPINA1/PI3/RPS6KA1/NLRP12/MAP3K5/FNIP1/PPM1F/SORL1/CR1/LYN/CFLAR/DNAJB6/MMP9/MEFV/RAF1 | 23 |
| BP | GO:0030100 | regulation of endocytosis | 15/421 | 229/17653 | 0.000413 | 0.017312 | 0.014665 | FLOT1/UNC119/RAB20/F2RL1/SIRPB1/PICALM/CD300LF/CD300A/PACSIN2/SLC11A1/SIRPA/TLR2/IL1B/HCK/PLCG2 | 15 |
| BP | GO:0072574 | hepatocyte proliferation | 4/421 | 16/17653 | 0.000462 | 0.018957 | 0.016058 | SULF2/CEBPB/RPS6KA1/CFLAR | 4 |
| BP | GO:0072575 | epithelial cell proliferation involved in liver morphogenesis | 4/421 | 16/17653 | 0.000462 | 0.018957 | 0.016058 | SULF2/CEBPB/RPS6KA1/CFLAR | 4 |
| BP | GO:0002675 | positive regulation of acute inflammatory response | 5/421 | 28/17653 | 0.000471 | 0.018957 | 0.016058 | PTGS2/ADAM8/FFAR2/IL1B/OSM | 5 |
| BP | GO:0072606 | interleukin-8 secretion | 5/421 | 28/17653 | 0.000471 | 0.018957 | 0.016058 | F2RL1/TLR1/TLR6/FFAR2/TLR2 | 5 |
| BP | GO:0050867 | positive regulation of cell activation | 20/421 | 363/17653 | 0.000479 | 0.019094 | 0.016174 | TNFSF14/GAB2/F2RL1/TFRC/SIRPB1/PELI1/TLR4/ADAM8/RPS6KA1/LGALS1/FLOT2/BCL6/PTAFR/TLR6/SIRPA/IRS2/PAK1/IL1B/STAT5B/LYN | 20 |
| BP | GO:0009612 | response to mechanical stimulus | 14/421 | 209/17653 | 0.000511 | 0.020184 | 0.017098 | TNFSF14/TLR8/FOS/PTGS2/TNFRSF1A/TLR4/TLR5/MAP2K4/HDAC4/MAP3K2/IL1B/KCNJ2/SLC9A1/RAF1 | 14 |
| BP | GO:0071375 | cellular response to peptide hormone stimulus | 18/421 | 312/17653 | 0.000531 | 0.020778 | 0.0176 | STAT3/RNASEL/ADCY4/PXN/CDK4/GHRL/NDEL1/ATP6V0B/IRS2/PAK1/PRKDC/IL1B/STAT5B/LYN/ATP6V1B2/CFLAR/ATP6V0D1/SLC9A1 | 18 |
| BP | GO:0006826 | iron ion transport | 7/421 | 60/17653 | 0.000542 | 0.021032 | 0.017816 | TFRC/PICALM/ATP6V0B/SLC11A1/STEAP4/ATP6V1B2/ATP6V0D1 | 7 |
| BP | GO:0032728 | positive regulation of interferon-beta production | 5/421 | 29/17653 | 0.000558 | 0.021432 | 0.018155 | TLR8/FLOT1/RNF135/TLR4/TLR2 | 5 |
| BP | GO:0010832 | negative regulation of myotube differentiation | 4/421 | 17/17653 | 0.000593 | 0.022183 | 0.018791 | NOTCH1/HDAC4/NOV/CFLAR | 4 |
| BP | GO:0034143 | regulation of toll-like receptor 4 signaling pathway | 4/421 | 17/17653 | 0.000593 | 0.022183 | 0.018791 | F2RL1/PELI1/LILRA2/LYN | 4 |
| BP | GO:0072576 | liver morphogenesis | 4/421 | 17/17653 | 0.000593 | 0.022183 | 0.018791 | SULF2/CEBPB/RPS6KA1/CFLAR | 4 |
| BP | GO:0030888 | regulation of B cell proliferation | 7/421 | 61/17653 | 0.0006 | 0.022249 | 0.018847 | TFRC/PELI1/CD300A/TLR4/BCL6/IRS2/LYN | 7 |
| BP | GO:0002683 | negative regulation of immune system process | 22/421 | 426/17653 | 0.000611 | 0.022335 | 0.018919 | F2RL1/PELI1/CD300LF/CEBPB/CD300A/PGLYRP1/TLR4/NLRP12/LILRA2/NOTCH1/SEC14L1/BCL6/TLR6/CR1/TRIB1/PRKDC/PADI2/STAT5B/LYN/LILRB3/NOV/CDK6 | 22 |
| BP | GO:0006937 | regulation of muscle contraction | 12/421 | 165/17653 | 0.000613 | 0.022335 | 0.018919 | PTGS2/GHRL/MYBPH/PTAFR/DOCK5/HDAC4/MYBPC3/PROK2/KCNJ2/DSC2/PDE4B/SLC9A1 | 12 |
| BP | GO:0043434 | response to peptide hormone | 22/421 | 427/17653 | 0.00063 | 0.022746 | 0.019268 | STAT3/RNASEL/PTGS2/ADCY4/PXN/CDK4/GHRL/NDEL1/ADM/ATP6V0B/IRS2/PAK1/PRKDC/TLR2/IL1B/STAT5B/LYN/ATP6V1B2/CFLAR/ATP6V0D1/SLC9A1/IQGAP1 | 22 |
| BP | GO:0002279 | mast cell activation involved in immune response | 6/421 | 45/17653 | 0.000658 | 0.023361 | 0.019788 | GAB2/SNAP23/LAT2/CD300A/PIK3CD/LYN | 6 |
| BP | GO:0043303 | mast cell degranulation | 6/421 | 45/17653 | 0.000658 | 0.023361 | 0.019788 | GAB2/SNAP23/LAT2/CD300A/PIK3CD/LYN | 6 |
| BP | GO:0070542 | response to fatty acid | 8/421 | 81/17653 | 0.000689 | 0.024239 | 0.020532 | PTGS2/DGAT2/ACSL1/CDK4/PTAFR/FFAR2/TLR2/PDK3 | 8 |
| BP | GO:0043281 | regulation of cysteine-type endopeptidase activity involved in apoptotic process | 14/421 | 216/17653 | 0.000708 | 0.024699 | 0.020922 | TNFSF14/APAF1/PLAUR/TNFRSF10C/PTGS2/RPS6KA1/NLRP12/MAP3K5/FNIP1/PPM1F/CFLAR/DNAJB6/MMP9/RAF1 | 14 |
| BP | GO:0051051 | negative regulation of transport | 23/421 | 460/17653 | 0.00072 | 0.024918 | 0.021108 | TLR8/UNC119/F2RL1/PTGS2/PICALM/CD300LF/CD300A/OAZ2/GHRL/PACSIN2/PIM3/NLRP12/NOTCH1/REM2/MIDN/TLR6/IRS2/TLR2/IL1B/OSM/NOV/HSPA1A/MMP9 | 23 |
| BP | GO:0043406 | positive regulation of MAP kinase activity | 16/421 | 268/17653 | 0.000741 | 0.025279 | 0.021414 | FPR1/C5AR1/MAP3K3/GHRL/TLR4/ADAM8/MAP3K5/MAP2K4/TLR6/MAP3K2/PAK1/PROK2/IL1B/IQGAP1/TGFA/RAF1 | 16 |
| BP | GO:0032612 | interleukin-1 production | 8/421 | 82/17653 | 0.000747 | 0.025279 | 0.021414 | TLR8/F2RL1/GHRL/TLR4/NLRP12/TLR6/IL1B/MEFV | 8 |
| BP | GO:0002320 | lymphoid progenitor cell differentiation | 4/421 | 18/17653 | 0.000748 | 0.025279 | 0.021414 | FLCN/NOTCH1/FNIP1/PRKDC | 4 |
| BP | GO:0006691 | leukotriene metabolic process | 5/421 | 31/17653 | 0.000767 | 0.025509 | 0.021608 | ALOX5/CYP4F3/MGST3/TLR2/ALOX5AP | 5 |
| BP | GO:0034142 | toll-like receptor 4 signaling pathway | 5/421 | 31/17653 | 0.000767 | 0.025509 | 0.021608 | F2RL1/PELI1/TLR4/LILRA2/LYN | 5 |
| BP | GO:0046330 | positive regulation of JNK cascade | 10/421 | 124/17653 | 0.000778 | 0.02567 | 0.021745 | F2RL1/RASSF2/NCF1/TLR4/MAP3K5/MAP2K4/TLR6/MAP3K2/PAK1/IL1B | 10 |
| BP | GO:0001933 | negative regulation of protein phosphorylation | 22/421 | 435/17653 | 0.000804 | 0.026322 | 0.022297 | SH3BP5L/RNF149/F2RL1/RASSF2/CDK4/CD300A/TLR4/NLRP12/FLCN/STRAP/PPM1F/SORL1/TRIB1/PRKDC/IL1B/PIN1/PREX1/LYN/DUSP1/CDK6/MVP/FAM129A | 22 |
| BP | GO:0002696 | positive regulation of leukocyte activation | 19/421 | 351/17653 | 0.000813 | 0.026397 | 0.02236 | TNFSF14/GAB2/F2RL1/TFRC/SIRPB1/PELI1/TLR4/ADAM8/LGALS1/FLOT2/BCL6/PTAFR/TLR6/SIRPA/IRS2/PAK1/IL1B/STAT5B/LYN | 19 |
| BP | GO:0001818 | negative regulation of cytokine production | 15/421 | 245/17653 | 0.000832 | 0.026651 | 0.022576 | RPS6KA5/TLR8/BCL3/F2RL1/GHRL/RNF135/UBE2L6/PGLYRP1/TLR4/NLRP12/BCL6/SLC11A1/TLR6/PIN1/MEFV | 15 |
| BP | GO:0002448 | mast cell mediated immunity | 6/421 | 47/17653 | 0.000833 | 0.026651 | 0.022576 | GAB2/SNAP23/LAT2/CD300A/PIK3CD/LYN | 6 |
| BP | GO:1903426 | regulation of reactive oxygen species biosynthetic process | 8/421 | 84/17653 | 0.000877 | 0.027841 | 0.023583 | STAT3/PTGS2/TLR4/TLR5/TLR6/HDAC4/IL1B/CFLAR | 8 |
| BP | GO:0010574 | regulation of vascular endothelial growth factor production | 5/421 | 32/17653 | 0.000892 | 0.028087 | 0.023792 | SULF2/PTGS2/C5AR1/IL1B/HPSE | 5 |
| BP | GO:0071902 | positive regulation of protein serine/threonine kinase activity | 19/421 | 355/17653 | 0.00093 | 0.02844 | 0.024091 | FPR1/RALB/ACSL1/C5AR1/MAP3K3/GHRL/TLR4/ADAM8/MAP3K5/CDK5R1/MAP2K4/TLR6/MAP3K2/PAK1/PROK2/IL1B/IQGAP1/TGFA/RAF1 | 19 |
| BP | GO:0002902 | regulation of B cell apoptotic process | 4/421 | 19/17653 | 0.00093 | 0.02844 | 0.024091 | BCL6/FNIP1/IRS2/LYN | 4 |
| BP | GO:0042533 | tumor necrosis factor biosynthetic process | 4/421 | 19/17653 | 0.00093 | 0.02844 | 0.024091 | BCL3/GHRL/TLR4/TLR1 | 4 |
| BP | GO:0042534 | regulation of tumor necrosis factor biosynthetic process | 4/421 | 19/17653 | 0.00093 | 0.02844 | 0.024091 | BCL3/GHRL/TLR4/TLR1 | 4 |
| BP | GO:2000116 | regulation of cysteine-type endopeptidase activity | 15/421 | 249/17653 | 0.000981 | 0.029777 | 0.025224 | TNFSF14/APAF1/PLAUR/TNFRSF10C/PTGS2/RPS6KA1/NLRP12/MAP3K5/FNIP1/PPM1F/CFLAR/DNAJB6/MMP9/MEFV/RAF1 | 15 |
| BP | GO:0032874 | positive regulation of stress-activated MAPK cascade | 11/421 | 151/17653 | 0.001009 | 0.030418 | 0.025767 | F2RL1/RASSF2/MAP3K3/NCF1/TLR4/MAP3K5/MAP2K4/TLR6/MAP3K2/PAK1/IL1B | 11 |
| BP | GO:1902624 | positive regulation of neutrophil migration | 5/421 | 33/17653 | 0.001031 | 0.030839 | 0.026123 | C5AR1/ADAM8/MOSPD2/CXCL1/DAPK2 | 5 |
| BP | GO:0045806 | negative regulation of endocytosis | 6/421 | 49/17653 | 0.001043 | 0.030982 | 0.026244 | UNC119/PICALM/CD300LF/CD300A/PACSIN2/TLR2 | 6 |
| BP | GO:0032418 | lysosome localization | 7/421 | 67/17653 | 0.001059 | 0.031147 | 0.026384 | GAB2/SNAP23/LAT2/CD300A/NDEL1/PIK3CD/LYN | 7 |
| BP | GO:0010951 | negative regulation of endopeptidase activity | 15/421 | 251/17653 | 0.001063 | 0.031147 | 0.026384 | TNFSF14/PLAUR/TIMP2/TNFRSF10C/PTGS2/PICALM/SERPINA1/PI3/RPS6KA1/FNIP1/SORL1/CR1/DNAJB6/MMP9/RAF1 | 15 |
| BP | GO:2000773 | negative regulation of cellular senescence | 4/421 | 20/17653 | 0.001141 | 0.03319 | 0.028115 | MAP3K3/BCL6/PRKDC/CDK6 | 4 |
| BP | GO:0050920 | regulation of chemotaxis | 13/421 | 202/17653 | 0.001158 | 0.033449 | 0.028334 | TNFSF14/STX3/F2RL1/C5AR1/IL6R/NOTCH1/PPM1F/MOSPD2/PADI2/LYN/NOV/CXCL1/DAPK2 | 13 |
| BP | GO:0010573 | vascular endothelial growth factor production | 5/421 | 34/17653 | 0.001185 | 0.034 | 0.028801 | SULF2/PTGS2/C5AR1/IL1B/HPSE | 5 |
| BP | GO:0032868 | response to insulin | 15/421 | 254/17653 | 0.001198 | 0.034021 | 0.028818 | RNASEL/CDK4/NDEL1/ADM/ATP6V0B/IRS2/PAK1/PRKDC/TLR2/IL1B/LYN/ATP6V1B2/CFLAR/ATP6V0D1/SLC9A1 | 15 |
| BP | GO:0007159 | leukocyte cell-cell adhesion | 18/421 | 335/17653 | 0.001202 | 0.034021 | 0.028818 | TNFSF14/TFRC/SIRPB1/PELI1/PECAM1/CEBPB/CD300A/ADAM8/LGALS1/FLOT2/BCL6/PTAFR/SIRPA/PAK1/IL1B/STAT5B/LYN/ITGA5 | 18 |
| BP | GO:0072593 | reactive oxygen species metabolic process | 15/421 | 256/17653 | 0.001295 | 0.036403 | 0.030836 | STAT3/F2RL1/PTGS2/NCF1/TLR4/TLR5/HK2/TLR6/HDAC4/TLR2/IL1B/PDK3/PREX1/CFLAR/NCF2 | 15 |
| BP | GO:0002768 | immune response-regulating cell surface receptor signaling pathway | 23/421 | 482/17653 | 0.001339 | 0.037404 | 0.031684 | RPS6KA5/FPR1/GAB2/FCGR2A/FOS/C5AR1/LAT2/CD300A/FCGRT/LILRA2/ICAM3/MAP2K4/FFAR2/PIK3CD/CR1/PAK1/CLEC4E/LYN/PDE4B/HCK/NFAM1/RAF1/PLCG2 | 23 |
| BP | GO:2000117 | negative regulation of cysteine-type endopeptidase activity | 9/421 | 111/17653 | 0.001357 | 0.037648 | 0.031891 | TNFSF14/PLAUR/TNFRSF10C/PTGS2/RPS6KA1/FNIP1/DNAJB6/MMP9/RAF1 | 9 |
| BP | GO:0001765 | membrane raft assembly | 3/421 | 10/17653 | 0.001426 | 0.038895 | 0.032947 | FLOT1/PACSIN2/FLOT2 | 3 |
| BP | GO:0072672 | neutrophil extravasation | 3/421 | 10/17653 | 0.001426 | 0.038895 | 0.032947 | PECAM1/ADAM8/PIK3CD | 3 |
| BP | GO:0050766 | positive regulation of phagocytosis | 6/421 | 52/17653 | 0.00143 | 0.038895 | 0.032947 | F2RL1/SIRPB1/CD300LF/SLC11A1/SIRPA/IL1B | 6 |
| BP | GO:0050708 | regulation of protein secretion | 21/421 | 426/17653 | 0.001442 | 0.038961 | 0.033003 | TLR8/CACNA1E/F2RL1/IL1RAP/GHRL/TLR4/PIM3/ADAM8/NLRP12/TLR5/IL17RA/TLR1/MIDN/TLR6/FFAR2/IRS2/CLEC4E/TLR2/IL1B/LYN/NOV | 21 |
| BP | GO:0030203 | glycosaminoglycan metabolic process | 11/421 | 158/17653 | 0.001458 | 0.039148 | 0.033161 | ABCC5/B4GALT5/PGLYRP1/ST3GAL2/ST3GAL4/IDS/IL1B/HPSE/CHSY1/SLC9A1/NDST1 | 11 |
| BP | GO:0030316 | osteoclast differentiation | 8/421 | 91/17653 | 0.001481 | 0.039335 | 0.03332 | GAB2/FOS/TFRC/RASSF2/CEBPB/TLR4/LILRB3/SBNO2 | 8 |
| BP | GO:0051222 | positive regulation of protein transport | 21/421 | 427/17653 | 0.001484 | 0.039335 | 0.03332 | TLR8/F2RL1/PTGS2/IL1RAP/OAZ2/GHRL/TLR4/ADAM8/NLRP12/IL17RA/CDK5R1/TLR1/FFAR2/SORL1/IRS2/SH3GLB1/PAK1/CLEC4E/TLR2/IL1B/GSK3B | 21 |
| BP | GO:1903531 | negative regulation of secretion by cell | 12/421 | 183/17653 | 0.00151 | 0.039795 | 0.033709 | TLR8/F2RL1/CD300A/GHRL/PIM3/NLRP12/NOTCH1/MIDN/TLR6/IL1B/OSM/NOV | 12 |
| BP | GO:0060416 | response to growth hormone | 5/421 | 36/17653 | 0.001544 | 0.040426 | 0.034244 | STAT3/PXN/GHRL/STAT5B/LYN | 5 |
| BP | GO:1903428 | positive regulation of reactive oxygen species biosynthetic process | 6/421 | 53/17653 | 0.001581 | 0.041124 | 0.034835 | PTGS2/TLR4/TLR5/TLR6/HDAC4/IL1B | 6 |
| BP | GO:1903510 | mucopolysaccharide metabolic process | 9/421 | 114/17653 | 0.001636 | 0.042293 | 0.035826 | ABCC5/B4GALT5/ST3GAL2/ST3GAL4/IDS/IL1B/CHSY1/SLC9A1/NDST1 | 9 |
| BP | GO:0060627 | regulation of vesicle-mediated transport | 23/421 | 490/17653 | 0.001658 | 0.042602 | 0.036087 | CHMP2A/FLOT1/UNC119/GAB2/RAB20/F2RL1/SIRPB1/RALB/PICALM/CD300LF/CD300A/PACSIN2/NOTCH1/SLC11A1/PTAFR/SIRPA/SORL1/TLR2/IL1B/TBC1D14/LYN/HCK/PLCG2 | 23 |
| BP | GO:0014812 | muscle cell migration | 8/421 | 93/17653 | 0.001703 | 0.043494 | 0.036843 | PLEKHO1/DOCK5/SORL1/HDAC4/PAK1/TRIB1/NOV/IQGAP1 | 8 |
| BP | GO:0050730 | regulation of peptidyl-tyrosine phosphorylation | 14/421 | 237/17653 | 0.00172 | 0.043527 | 0.036871 | STAT3/UNC119/SH3BP5L/TNFRSF1A/CD300A/GHRL/IL6R/NCF1/OSM/LYN/ITGA5/MVP/IQGAP1/TGFA | 14 |
| BP | GO:0031099 | regeneration | 12/421 | 186/17653 | 0.001733 | 0.043527 | 0.036871 | SULF2/CDK4/C5AR1/CEBPB/NDEL1/ADM/NOTCH1/ULK1/NOV/CFLAR/IGF2R/NINJ1 | 12 |
| BP | GO:0051154 | negative regulation of striated muscle cell differentiation | 5/421 | 37/17653 | 0.001751 | 0.043527 | 0.036871 | NOTCH1/HDAC4/PAK1/NOV/CFLAR | 5 |
| BP | GO:0010466 | negative regulation of peptidase activity | 15/421 | 264/17653 | 0.001753 | 0.043527 | 0.036871 | TNFSF14/PLAUR/TIMP2/TNFRSF10C/PTGS2/PICALM/SERPINA1/PI3/RPS6KA1/FNIP1/SORL1/CR1/DNAJB6/MMP9/RAF1 | 15 |
| BP | GO:0032481 | positive regulation of type I interferon production | 7/421 | 73/17653 | 0.001756 | 0.043527 | 0.036871 | TLR8/FLOT1/RNF135/TLR4/PRKDC/TLR2/PLCG2 | 7 |
| BP | GO:0002285 | lymphocyte activation involved in immune response | 11/421 | 162/17653 | 0.00178 | 0.043833 | 0.03713 | BCL3/STAT3/F2RL1/TFRC/PGLYRP1/TLR4/LGALS1/BCL6/SLC11A1/CLEC4E/PLCG2 | 11 |
| BP | GO:0050714 | positive regulation of protein secretion | 14/421 | 238/17653 | 0.001789 | 0.043833 | 0.03713 | TLR8/F2RL1/IL1RAP/GHRL/TLR4/ADAM8/NLRP12/IL17RA/TLR1/FFAR2/IRS2/CLEC4E/TLR2/IL1B | 14 |
| BP | GO:0042742 | defense response to bacterium | 16/421 | 293/17653 | 0.001875 | 0.045677 | 0.038692 | BCL3/F2RL1/HIST1H2BC/TNFRSF1A/HIST1H2BE/C5AR1/CEBPB/IL6R/PGLYRP1/TLR4/TLR5/TLR1/SLC11A1/TLR6/CLEC4E/TLR2 | 16 |
| BP | GO:0032642 | regulation of chemokine production | 7/421 | 74/17653 | 0.001901 | 0.04586 | 0.038847 | F2RL1/IL6R/TLR4/FFAR2/TLR2/IL1B/MEFV | 7 |
| BP | GO:0090084 | negative regulation of inclusion body assembly | 3/421 | 11/17653 | 0.001927 | 0.04586 | 0.038847 | SORL1/HSPA1A/DNAJB6 | 3 |
| BP | GO:1902992 | negative regulation of amyloid precursor protein catabolic process | 3/421 | 11/17653 | 0.001927 | 0.04586 | 0.038847 | PICALM/FLOT2/SORL1 | 3 |
| BP | GO:2000786 | positive regulation of autophagosome assembly | 3/421 | 11/17653 | 0.001927 | 0.04586 | 0.038847 | RALB/ULK1/SH3GLB1 | 3 |
| BP | GO:1902991 | regulation of amyloid precursor protein catabolic process | 4/421 | 23/17653 | 0.001971 | 0.046532 | 0.039417 | PICALM/FLOT2/SORL1/LYN | 4 |
| BP | GO:1902622 | regulation of neutrophil migration | 5/421 | 38/17653 | 0.001977 | 0.046532 | 0.039417 | C5AR1/ADAM8/MOSPD2/CXCL1/DAPK2 | 5 |
| BP | GO:0006936 | muscle contraction | 18/421 | 352/17653 | 0.002076 | 0.048586 | 0.041156 | SULF2/PTGS2/COL4A3BP/PXN/GHRL/MYBPH/NDUFS6/TPCN2/PTAFR/DOCK5/HDAC4/MYBPC3/PROK2/KCNJ2/DSC2/PDE4B/SLC9A1/DYSF | 18 |
| BP | GO:0010810 | regulation of cell-substrate adhesion | 12/421 | 191/17653 | 0.002164 | 0.050372 | 0.042669 | LGALS1/NOTCH1/BCL6/DOCK5/PPM1F/PREX1/GSK3B/ITGA5/SLC9A1/CDK6/IQGAP1/NINJ1 | 12 |
| BP | GO:0070302 | regulation of stress-activated protein kinase signaling cascade | 13/421 | 217/17653 | 0.002195 | 0.050801 | 0.043033 | F2RL1/RASSF2/MAP3K3/EPHB1/NCF1/TLR4/MAP3K5/MAP2K4/TLR6/MAP3K2/PAK1/IL1B/LYN | 13 |
| BP | GO:0015682 | ferric iron transport | 5/421 | 39/17653 | 0.002224 | 0.050916 | 0.04313 | TFRC/ATP6V0B/STEAP4/ATP6V1B2/ATP6V0D1 | 5 |
| BP | GO:0072512 | trivalent inorganic cation transport | 5/421 | 39/17653 | 0.002224 | 0.050916 | 0.04313 | TFRC/ATP6V0B/STEAP4/ATP6V1B2/ATP6V0D1 | 5 |
| BP | GO:0001783 | B cell apoptotic process | 4/421 | 24/17653 | 0.002321 | 0.052468 | 0.044445 | BCL6/FNIP1/IRS2/LYN | 4 |
| BP | GO:2000778 | positive regulation of interleukin-6 secretion | 4/421 | 24/17653 | 0.002321 | 0.052468 | 0.044445 | TLR8/F2RL1/IL1RAP/IL1B | 4 |
| BP | GO:0050709 | negative regulation of protein secretion | 9/421 | 120/17653 | 0.00233 | 0.052468 | 0.044445 | TLR8/F2RL1/GHRL/PIM3/NLRP12/MIDN/TLR6/IL1B/NOV | 9 |
| BP | GO:0002703 | regulation of leukocyte mediated immunity | 11/421 | 168/17653 | 0.00237 | 0.052967 | 0.044867 | GAB2/F2RL1/TFRC/CD300A/TLR4/BCL6/PTAFR/CR1/IL1B/STAT5B/LYN | 11 |
| BP | GO:0032147 | activation of protein kinase activity | 17/421 | 328/17653 | 0.002377 | 0.052967 | 0.044867 | FPR1/ADCY4/C5AR1/MAP3K3/CD300A/GHRL/TLR4/MAP3K5/MAP2K4/SLC11A1/TLR6/MAP3K2/PAK1/PROK2/IL1B/TGFA/RAF1 | 17 |
| BP | GO:0031100 | animal organ regeneration | 7/421 | 77/17653 | 0.00239 | 0.052969 | 0.044869 | SULF2/CDK4/C5AR1/CEBPB/ADM/NOTCH1/IGF2R | 7 |
| BP | GO:0030183 | B cell differentiation | 9/421 | 121/17653 | 0.002465 | 0.053128 | 0.045004 | BCL3/LGALS1/BCL6/FNIP1/HDAC4/PRKDC/STAT5B/NFAM1/PLCG2 | 9 |
| BP | GO:0046328 | regulation of JNK cascade | 11/421 | 169/17653 | 0.002483 | 0.053128 | 0.045004 | F2RL1/RASSF2/EPHB1/NCF1/TLR4/MAP3K5/MAP2K4/TLR6/MAP3K2/PAK1/IL1B | 11 |
| BP | GO:0045980 | negative regulation of nucleotide metabolic process | 6/421 | 58/17653 | 0.002522 | 0.053128 | 0.045004 | STAT3/P2RY13/CDA/FLCN/EGLN1/HDAC4 | 6 |
| BP | GO:0001660 | fever generation | 3/421 | 12/17653 | 0.002524 | 0.053128 | 0.045004 | PTGS2/IL1RN/IL1B | 3 |
| BP | GO:0002430 | complement receptor mediated signaling pathway | 3/421 | 12/17653 | 0.002524 | 0.053128 | 0.045004 | FPR1/C5AR1/CR1 | 3 |
| BP | GO:0014831 | gastro-intestinal system smooth muscle contraction | 3/421 | 12/17653 | 0.002524 | 0.053128 | 0.045004 | SULF2/GHRL/PTAFR | 3 |
| BP | GO:0014842 | regulation of skeletal muscle satellite cell proliferation | 3/421 | 12/17653 | 0.002524 | 0.053128 | 0.045004 | STAT3/EPHB1/CFLAR | 3 |
| BP | GO:0044090 | positive regulation of vacuole organization | 3/421 | 12/17653 | 0.002524 | 0.053128 | 0.045004 | RALB/ULK1/SH3GLB1 | 3 |
| BP | GO:0045414 | regulation of interleukin-8 biosynthetic process | 3/421 | 12/17653 | 0.002524 | 0.053128 | 0.045004 | TLR8/BCL3/TLR4 | 3 |
| BP | GO:1900225 | regulation of NLRP3 inflammasome complex assembly | 3/421 | 12/17653 | 0.002524 | 0.053128 | 0.045004 | TLR4/TLR6/MEFV | 3 |
| BP | GO:1903037 | regulation of leukocyte cell-cell adhesion | 16/421 | 302/17653 | 0.00254 | 0.053204 | 0.045068 | TNFSF14/TFRC/SIRPB1/PELI1/CEBPB/CD300A/ADAM8/LGALS1/FLOT2/BCL6/PTAFR/SIRPA/PAK1/IL1B/STAT5B/LYN | 16 |
| BP | GO:0048659 | smooth muscle cell proliferation | 10/421 | 146/17653 | 0.002662 | 0.055484 | 0.047 | PTGS2/IL6R/MAP3K5/PTAFR/HDAC4/PAK1/TRIB1/PRKDC/NOV/MMP9 | 10 |
| BP | GO:1905048 | regulation of metallopeptidase activity | 4/421 | 25/17653 | 0.002712 | 0.056231 | 0.047632 | STAT3/TIMP2/PICALM/SORL1 | 4 |
| BP | GO:0002687 | positive regulation of leukocyte migration | 9/421 | 123/17653 | 0.002755 | 0.056574 | 0.047923 | TNFSF14/F2RL1/C5AR1/IL6R/ADAM8/PTAFR/MOSPD2/CXCL1/DAPK2 | 9 |
| BP | GO:0032479 | regulation of type I interferon production | 9/421 | 123/17653 | 0.002755 | 0.056574 | 0.047923 | TLR8/FLOT1/RNF135/UBE2L6/TLR4/PRKDC/TLR2/PIN1/PLCG2 | 9 |
| BP | GO:1903706 | regulation of hemopoiesis | 21/421 | 450/17653 | 0.002781 | 0.05658 | 0.047927 | STAT3/FOS/RASSF2/CEBPB/PGLYRP1/TLR4/ADAM8/FLCN/HIST1H3D/NOTCH1/BCL6/FNIP1/CR1/TRIB1/STAT5B/LYN/LILRB3/CSF3R/HSPA1A/NFAM1/CDK6 | 21 |
| BP | GO:0043122 | regulation of I-kappaB kinase/NF-kappaB signaling | 13/421 | 223/17653 | 0.002782 | 0.05658 | 0.047927 | CANT1/F2RL1/PELI1/TNFRSF1A/MAP3K3/LITAF/NLRP12/USP10/LGALS1/TLR6/IL1B/PELI2/CFLAR | 13 |
| BP | GO:0000187 | activation of MAPK activity | 10/421 | 147/17653 | 0.002798 | 0.056614 | 0.047956 | FPR1/C5AR1/GHRL/TLR4/MAP3K5/MAP2K4/MAP3K2/PROK2/IL1B/TGFA | 10 |
| BP | GO:1903039 | positive regulation of leukocyte cell-cell adhesion | 13/421 | 224/17653 | 0.002892 | 0.058238 | 0.049332 | TNFSF14/TFRC/SIRPB1/ADAM8/LGALS1/FLOT2/BCL6/PTAFR/SIRPA/PAK1/IL1B/STAT5B/LYN | 13 |
| BP | GO:0032606 | type I interferon production | 9/421 | 124/17653 | 0.00291 | 0.058243 | 0.049337 | TLR8/FLOT1/RNF135/UBE2L6/TLR4/PRKDC/TLR2/PIN1/PLCG2 | 9 |
| BP | GO:0031331 | positive regulation of cellular catabolic process | 17/421 | 335/17653 | 0.002954 | 0.058243 | 0.049337 | PFKFB4/ABHD5/RALB/TP53INP2/UPF1/ADAM8/FLCN/HK2/ULK1/IRS2/SH3GLB1/TRIB1/IL1B/GSK3B/DTX3L/HSPA1A/MEFV | 17 |
| BP | GO:0009896 | positive regulation of catabolic process | 19/421 | 393/17653 | 0.002959 | 0.058243 | 0.049337 | PFKFB4/ABHD5/RALB/TP53INP2/UPF1/OAZ2/ADAM8/FLCN/HK2/ULK1/SORL1/IRS2/SH3GLB1/TRIB1/IL1B/GSK3B/DTX3L/HSPA1A/MEFV | 19 |
| BP | GO:0046427 | positive regulation of JAK-STAT cascade | 7/421 | 80/17653 | 0.002971 | 0.058243 | 0.049337 | STAT3/TNFRSF1A/CD300A/IL6R/NOTCH1/OSM/LYN | 7 |
| BP | GO:0043409 | negative regulation of MAPK cascade | 11/421 | 173/17653 | 0.002975 | 0.058243 | 0.049337 | RNF149/F2RL1/CD300A/TLR4/NLRP12/FLCN/SORL1/IL1B/PIN1/LYN/DUSP1 | 11 |
| BP | GO:2000377 | regulation of reactive oxygen species metabolic process | 11/421 | 173/17653 | 0.002975 | 0.058243 | 0.049337 | STAT3/F2RL1/PTGS2/TLR4/TLR5/HK2/TLR6/HDAC4/IL1B/PDK3/CFLAR | 11 |
| BP | GO:0032609 | interferon-gamma production | 8/421 | 102/17653 | 0.003048 | 0.058352 | 0.049429 | TLR8/BCL3/F2RL1/PGLYRP1/TLR4/SLC11A1/IL1B/PDE4B | 8 |
| BP | GO:0030890 | positive regulation of B cell proliferation | 5/421 | 42/17653 | 0.0031 | 0.058352 | 0.049429 | TFRC/PELI1/TLR4/BCL6/IRS2 | 5 |
| BP | GO:0045429 | positive regulation of nitric oxide biosynthetic process | 5/421 | 42/17653 | 0.0031 | 0.058352 | 0.049429 | PTGS2/TLR4/TLR5/TLR6/IL1B | 5 |
| BP | GO:1904407 | positive regulation of nitric oxide metabolic process | 5/421 | 42/17653 | 0.0031 | 0.058352 | 0.049429 | PTGS2/TLR4/TLR5/TLR6/IL1B | 5 |
| BP | GO:0090344 | negative regulation of cell aging | 4/421 | 26/17653 | 0.003145 | 0.058352 | 0.049429 | MAP3K3/BCL6/PRKDC/CDK6 | 4 |
| BP | GO:1900745 | positive regulation of p38MAPK cascade | 4/421 | 26/17653 | 0.003145 | 0.058352 | 0.049429 | MAP3K3/NCF1/MAP3K5/IL1B | 4 |
| BP | GO:0007254 | JNK cascade | 12/421 | 200/17653 | 0.00316 | 0.058352 | 0.049429 | F2RL1/RASSF2/EPHB1/NCF1/TLR4/MAP3K5/MAP2K4/TLR6/MAP3K2/PAK1/TRIB1/IL1B | 12 |
| BP | GO:0002791 | regulation of peptide secretion | 21/421 | 455/17653 | 0.003164 | 0.058352 | 0.049429 | TLR8/CACNA1E/F2RL1/IL1RAP/GHRL/TLR4/PIM3/ADAM8/NLRP12/TLR5/IL17RA/TLR1/MIDN/TLR6/FFAR2/IRS2/CLEC4E/TLR2/IL1B/LYN/NOV | 21 |
| BP | GO:0051348 | negative regulation of transferase activity | 16/421 | 309/17653 | 0.003183 | 0.058352 | 0.049429 | SH3BP5L/CDK4/CD300A/MIDN/PPM1F/SORL1/IRS2/TRIB1/IL1B/PREX1/GSK3B/LYN/DUSP1/DTX3L/CDK6/MVP | 16 |
| BP | GO:0032602 | chemokine production | 7/421 | 81/17653 | 0.003187 | 0.058352 | 0.049429 | F2RL1/IL6R/TLR4/FFAR2/TLR2/IL1B/MEFV | 7 |
| BP | GO:0014841 | skeletal muscle satellite cell proliferation | 3/421 | 13/17653 | 0.003223 | 0.058352 | 0.049429 | STAT3/EPHB1/CFLAR | 3 |
| BP | GO:0014857 | regulation of skeletal muscle cell proliferation | 3/421 | 13/17653 | 0.003223 | 0.058352 | 0.049429 | STAT3/EPHB1/CFLAR | 3 |
| BP | GO:0042228 | interleukin-8 biosynthetic process | 3/421 | 13/17653 | 0.003223 | 0.058352 | 0.049429 | TLR8/BCL3/TLR4 | 3 |
| BP | GO:0044546 | NLRP3 inflammasome complex assembly | 3/421 | 13/17653 | 0.003223 | 0.058352 | 0.049429 | TLR4/TLR6/MEFV | 3 |
| BP | GO:1905049 | negative regulation of metallopeptidase activity | 3/421 | 13/17653 | 0.003223 | 0.058352 | 0.049429 | TIMP2/PICALM/SORL1 | 3 |
| BP | GO:0002220 | innate immune response activating cell surface receptor signaling pathway | 9/421 | 126/17653 | 0.00324 | 0.058352 | 0.049429 | RPS6KA5/LILRA2/ICAM3/FFAR2/PAK1/CLEC4E/LYN/RAF1/PLCG2 | 9 |
| BP | GO:0002792 | negative regulation of peptide secretion | 9/421 | 126/17653 | 0.00324 | 0.058352 | 0.049429 | TLR8/F2RL1/GHRL/PIM3/NLRP12/MIDN/TLR6/IL1B/NOV | 9 |
| BP | GO:0051346 | negative regulation of hydrolase activity | 21/421 | 456/17653 | 0.003246 | 0.058352 | 0.049429 | TNFSF14/PPP1R15A/PLAUR/TIMP2/TNFRSF10C/PTGS2/PICALM/SERPINA1/PI3/RPS6KA1/ELL/FNIP1/EGLN1/SORL1/CR1/GSK3B/DNAJB6/MMP9/F11R/IQGAP1/RAF1 | 21 |
| BP | GO:0010830 | regulation of myotube differentiation | 6/421 | 61/17653 | 0.00326 | 0.058352 | 0.049429 | TNFSF14/FLOT1/NOTCH1/HDAC4/NOV/CFLAR | 6 |
| BP | GO:0032835 | glomerulus development | 6/421 | 61/17653 | 0.00326 | 0.058352 | 0.049429 | BASP1/SULF2/PECAM1/NOTCH1/CFLAR/IQGAP1 | 6 |
| BP | GO:0032869 | cellular response to insulin stimulus | 12/421 | 201/17653 | 0.00329 | 0.058652 | 0.049683 | RNASEL/CDK4/NDEL1/ATP6V0B/IRS2/PAK1/PRKDC/IL1B/ATP6V1B2/CFLAR/ATP6V0D1/SLC9A1 | 12 |
