# Supplementary material for: A ten-genes-based diagnostic signature for atherosclerosis
Source: BMC Cardiovasc Disord. 2021 Oct 23;21:513. doi: 10.1186/s12872-021-02323-9 (PMC8540101; doi:10.1186/s12872-021-02323-9)
Supplement: Supplementary file 4 — Additional file 4. Table S3. Significantly enriched KEGG pathways. [file 12872_2021_2323_MOESM4_ESM.docx]

Table S3 Significantly enriched KEGG pathways.

| ID | Description | GeneRatio | BgRatio | pvalue | p.adjust | qvalue | geneID | Count |
| --- | --- | --- | --- | --- | --- | --- | --- | --- |
| sa05152 | Tuberculosis | 21/246 | 180/8039 | 1.18E-07 | 3.16E-05 | 2.39E-05 | 317/3920/2212/7132/1051/7099/533/2215/7096/1520/10333/3460/1378/3588/26253/7097/3553/9114/818/4802/5894 | 21 |
| hsa04380 | Osteoclast differentiation | 17/246 | 128/8039 | 3.08E-07 | 4.12E-05 | 3.11E-05 | 9846/2212/2353/10326/7132/653361/11027/2215/3454/4689/140885/3460/5293/3553/11025/4688/5336 | 17 |
| hsa05140 | Leishmaniasis | 12/246 | 77/8039 | 3.24E-06 | 0.000289 | 0.000218 | 2212/2353/5743/653361/7099/2215/4689/3460/1378/7097/3553/4688 | 12 |
| hsa04145 | Phagosome | 16/246 | 152/8039 | 1.51E-05 | 0.001009 | 0.000764 | 3920/2212/7037/653361/7099/533/2215/7846/1520/10333/4689/7097/526/3678/9114/4688 | 16 |
| hsa05162 | Measles | 15/246 | 138/8039 | 1.91E-05 | 0.001019 | 0.000772 | 317/6774/2353/4939/1019/7099/3454/5293/7097/3553/6777/2932/3303/1021/3310 | 15 |
| hsa05223 | Non-small cell lung cancer | 10/246 | 66/8039 | 2.80E-05 | 0.001246 | 0.000943 | 1871/6774/6256/1019/5293/6777/1021/7039/5894/5336 | 10 |
| hsa04668 | TNF signaling pathway | 13/246 | 112/8039 | 3.45E-05 | 0.001315 | 0.000995 | 9252/602/2353/5743/7132/1051/4217/6416/5293/3553/2919/8837/4318 | 13 |
| hsa05134 | Legionellosis | 9/246 | 57/8039 | 5.06E-05 | 0.00169 | 0.001279 | 317/7099/7100/1378/7097/3553/2919/3303/3310 | 9 |
| hsa05167 | Kaposi sarcoma-associated herpesvirus infection | 17/246 | 189/8039 | 6.34E-05 | 0.001879 | 0.001423 | 1871/6774/2353/5743/7132/1019/6416/3454/5293/57580/2932/4067/2919/3055/1021/5894/5336 | 17 |
| hsa05160 | Hepatitis C | 15/246 | 155/8039 | 7.50E-05 | 0.002002 | 0.001515 | 1871/317/23562/6774/6041/4939/7132/6256/1019/3454/5293/2932/8837/1021/5894 | 15 |
| hsa04666 | Fc gamma R-mediated phagocytosis | 11/246 | 93/8039 | 0.000117 | 0.002839 | 0.002149 | 3985/9846/2212/653361/2215/5293/5058/4067/3055/5894/5336 | 11 |
| hsa05120 | Epithelial cell signaling in Helicobacter pylori infection | 9/246 | 70/8039 | 0.000259 | 0.005664 | 0.004287 | 533/6416/5058/4067/526/2919/9114/50848/5336 | 9 |
| hsa05135 | Yersinia infection | 12/246 | 120/8039 | 0.00029 | 0.005664 | 0.004287 | 8935/2353/5829/7099/6195/6416/9815/5293/3553/2932/3678/4210 | 12 |
| hsa05418 | Fluid shear stress and atherosclerosis | 13/246 | 139/8039 | 0.000317 | 0.005664 | 0.004287 | 2353/7132/5175/653361/4217/6416/4259/7056/5293/3553/1843/4318/4688 | 13 |
| hsa04620 | Toll-like receptor signaling pathway | 11/246 | 104/8039 | 0.000318 | 0.005664 | 0.004287 | 51311/2353/7099/7100/7096/6416/10333/3454/5293/7097/3553 | 11 |
| hsa04142 | Lysosome | 12/246 | 128/8039 | 0.000526 | 0.008781 | 0.006647 | 410/3920/9516/3988/22901/533/6556/1520/3423/9114/4126/3482 | 12 |
| hsa04670 | Leukocyte transendothelial migration | 11/246 | 113/8039 | 0.000651 | 0.010228 | 0.007742 | 23562/5829/5175/653361/4689/5293/4318/4688/87/50848/5336 | 11 |
| hsa04662 | B cell receptor signaling pathway | 9/246 | 82/8039 | 0.000849 | 0.012589 | 0.009529 | 27071/2353/11027/5293/2932/4067/11025/5894/5336 | 9 |
| hsa04935 | Growth hormone synthesis, secretion and action | 11/246 | 119/8039 | 0.001005 | 0.013366 | 0.010117 | 6774/2353/196883/51738/6416/8660/5293/6777/2932/5894/5336 | 11 |
| hsa04664 | Fc epsilon RI signaling pathway | 8/246 | 68/8039 | 0.001038 | 0.013366 | 0.010117 | 9846/240/6416/5293/4067/241/5894/5336 | 8 |
| hsa05142 | Chagas disease (American trypanosomiasis) | 10/246 | 102/8039 | 0.001079 | 0.013366 | 0.010117 | 2353/7132/7099/6416/10333/3460/5293/7097/3553/8837 | 10 |
| hsa04012 | ErbB signaling pathway | 9/246 | 85/8039 | 0.001101 | 0.013366 | 0.010117 | 6416/5293/5058/6777/2932/7039/818/5894/5336 | 9 |
| hsa04630 | JAK-STAT signaling pathway | 13/246 | 162/8039 | 0.001357 | 0.015724 | 0.011903 | 6774/3570/1438/3454/3460/5293/3588/3597/6777/5008/1441/1439/5894 | 13 |
| hsa05219 | Bladder cancer | 6/246 | 41/8039 | 0.001413 | 0.015724 | 0.011903 | 1871/9252/1019/4318/23604/5894 | 6 |
| hsa05205 | Proteoglycans in cancer | 15/246 | 205/8039 | 0.001517 | 0.016206 | 0.012267 | 6774/5329/5829/7099/5293/5058/7097/10855/3678/4318/6548/8826/818/5894/5336 | 15 |
| hsa04066 | HIF-1 signaling pathway | 10/246 | 109/8039 | 0.001792 | 0.018399 | 0.013927 | 6774/7037/3570/7099/3099/54583/3460/5293/818/5336 | 10 |
| hsa04062 | Chemokine signaling pathway | 14/246 | 189/8039 | 0.001929 | 0.018795 | 0.014227 | 6774/196883/5829/653361/58191/5293/5058/57580/6777/2932/4067/2919/3055/5894 | 14 |
| hsa05214 | Glioma | 8/246 | 75/8039 | 0.001971 | 0.018795 | 0.014227 | 1871/1019/5293/1021/7039/818/5894/5336 | 8 |
| hsa05212 | Pancreatic cancer | 8/246 | 76/8039 | 0.002146 | 0.019758 | 0.014955 | 1871/6774/5899/1019/5293/1021/7039/5894 | 8 |
| hsa04140 | Autophagy - animal | 11/246 | 137/8039 | 0.003118 | 0.02775 | 0.021006 | 3920/89849/58476/29982/8408/8660/51100/5293/8837/23604/5894 | 11 |
| hsa04217 | Necroptosis | 12/246 | 159/8039 | 0.00343 | 0.029541 | 0.022361 | 27243/6774/7132/5836/7099/3454/3460/3553/6777/8837/8334/818 | 12 |
| hsa04060 | Cytokine-cytokine receptor interaction | 18/246 | 294/8039 | 0.003884 | 0.032303 | 0.024452 | 8740/8794/7132/3556/3557/3570/58191/23765/1438/3454/3460/3588/3597/3553/5008/2919/1441/1439 | 18 |
| hsa05161 | Hepatitis B | 12/246 | 162/8039 | 0.003992 | 0.032303 | 0.024452 | 1871/317/6774/2353/7099/6416/3454/5293/7097/6777/4318/5894 | 12 |
| hsa04064 | NF-kappa B signaling pathway | 9/246 | 104/8039 | 0.00445 | 0.034947 | 0.026453 | 8740/5743/7132/7099/3553/4067/2919/8837/5336 | 9 |
| hsa05211 | Renal cell carcinoma | 7/246 | 69/8039 | 0.004948 | 0.037745 | 0.028571 | 2271/201163/54583/5293/5058/7039/5894 | 7 |
| hsa05224 | Breast cancer | 11/246 | 147/8039 | 0.005334 | 0.039557 | 0.029943 | 1871/8648/2353/1019/4851/10023/5293/2932/23401/1021/5894 | 11 |
| hsa05235 | PD-L1 expression and PD-1 checkpoint pathway in cancer | 8/246 | 89/8039 | 0.005725 | 0.040379 | 0.030565 | 6774/2353/4215/7099/3460/5293/7097/5894 | 8 |
| hsa05164 | Influenza A | 12/246 | 170/8039 | 0.005862 | 0.040379 | 0.030565 | 317/6041/4939/7132/1019/7099/3454/3460/5293/3553/1021/5894 | 12 |
| hsa04932 | Non-alcoholic fatty liver disease (NAFLD) | 11/246 | 149/8039 | 0.005898 | 0.040379 | 0.030565 | 7132/6256/3570/9377/4726/4217/8660/5293/3553/2932/4728 | 11 |
| hsa05010 | Alzheimer disease | 19/246 | 334/8039 | 0.006702 | 0.044736 | 0.033863 | 317/5743/7132/22926/9377/4726/29982/4217/8851/8408/10023/7846/8660/5293/3553/2932/23401/4728/5894 | 19 |
| hsa05145 | Toxoplasmosis | 9/246 | 112/8039 | 0.007212 | 0.046964 | 0.035549 | 6774/240/7132/7099/3460/3588/7097/3303/3310 | 9 |
| hsa05323 | Rheumatoid arthritis | 8/246 | 93/8039 | 0.007439 | 0.04729 | 0.035796 | 2353/7099/533/7097/3553/526/2919/9114 | 8 |
| hsa04657 | IL-17 signaling pathway | 8/246 | 94/8039 | 0.007922 | 0.049192 | 0.037236 | 2353/5743/1051/23765/3553/2932/2919/4318 | 8 |
