# Supplementary material for: A ten-genes-based diagnostic signature for atherosclerosis
Source: BMC Cardiovasc Disord. 2021 Oct 23;21:513. doi: 10.1186/s12872-021-02323-9 (PMC8540101; doi:10.1186/s12872-021-02323-9)
Supplement: Supplementary file 5 — Additional file 5. Table S4. Top 50 in network string_interactions111.tsv ranked by MCC method. [file 12872_2021_2323_MOESM5_ESM.docx]

| Table S4 Top 50 in network string_interactions111.tsv ranked by MCC method. | | | |
| --- | --- | --- | --- |
| Rank | Name | Score |  |
| 1 | CXCL1 | 9.14E+08 |  |
| 2 | CXCR2 | 7.44E+08 |  |
| 3 | CXCR1 | 6.43E+08 |  |
| 4 | FPR2 | 5.76E+08 |  |
| 5 | C5AR1 | 5.68E+08 |  |
| 6 | FPR1 | 5.25E+08 |  |
| 7 | CXCL16 | 4.79E+08 |  |
| 8 | HCAR2 | 4.79E+08 |  |
| 9 | P2RY13 | 4.79E+08 |  |
| 10 | HCAR3 | 4.79E+08 |  |
| 11 | LPAR2 | 4.79E+08 |  |
| 12 | OXER1 | 4.79E+08 |  |
| 13 | ADCY4 | 4.79E+08 |  |
| 14 | TLR2 | 4.36E+08 |  |
| 15 | TLR4 | 4.36E+08 |  |
| 16 | TLR8 | 4.36E+08 |  |
| 17 | TLR1 | 4.30E+08 |  |
| 18 | IL1B | 3.90E+08 |  |
| 19 | MMP9 | 3.43E+08 |  |
| 20 | STAT3 | 3.00E+08 |  |
| 21 | TLR5 | 2.90E+08 |  |
| 22 | IL1RN | 2.48E+08 |  |
| 23 | PTGS2 | 2.41E+08 |  |
| 24 | TNFRSF1A | 1.68E+08 |  |
| 25 | TLR6 | 1.41E+08 |  |
| 26 | TREM1 | 1.32E+08 |  |
| 27 | FOS | 8.06E+07 |  |
| 28 | IL17RA | 1.52E+07 |  |
| 29 | CD93 | 7264593 |  |
| 30 | ADAM8 | 7257612 |  |
| 31 | SNAP23 | 7257608 |  |
| 32 | SLC2A3 | 7257605 |  |
| 33 | IL6R | 4723728 |  |
| 34 | SLC11A1 | 3681488 |  |
| 35 | PTAFR | 3640636 |  |
| 36 | CD300A | 3630451 |  |
| 37 | PLAUR | 3629758 |  |
| 38 | SIRPA | 3629524 |  |
| 39 | CEACAM3 | 3628856 |  |
| 40 | CKAP4 | 3628841 |  |
| 41 | DYNLL1 | 3628821 |  |
| 42 | MGAM | 3628813 |  |
| 43 | SLC15A4 | 3628805 |  |
| 44 | MOSPD2 | 3628804 |  |
| 45 | GPR97 | 3628800 |  |
| 46 | FCGR2A | 2235486 |  |
| 47 | CLEC4E | 1463052 |  |
| 48 | LYN | 859728 |  |
| 49 | PECAM1 | 385941 |  |
| 50 | PGLYRP1 | 126720 |  |
