# Supplementary material for: A ten-genes-based diagnostic signature for atherosclerosis
Source: BMC Cardiovasc Disord. 2021 Oct 23;21:513. doi: 10.1186/s12872-021-02323-9 (PMC8540101; doi:10.1186/s12872-021-02323-9)
Supplement: Supplementary file 6 — Additional file 6. Table S5. The potential relationships between the 10 identified genes and atherosclerosis. [file 12872_2021_2323_MOESM6_ESM.docx]

**Table S5. The potential relationships between the 10 identified genes and atherosclerosis.**

| **Gene** | **Potential associations with atherosclerosis** | **Reference** |
| --- | --- | --- |
| STAT3 | Abnormal activation of STAT3 regulates the progression of atherosclerosis through affecting endothelial cells, macrophages, inflammation, etc. | [1] |
| IL1RN | IL1RN is probably involved in atherogenesis by acting with IL-1, the interleukin which may be associated with the inflammatory mechanism of atherosclerosis formation. | [2] |
| C5AR1 | C5AR1 may be engaged in the progression of atherosclerosis lesions through the C5a-C5aR axis. | [3] |
| CXCL16 | CXCL16 may acts as a pro-inflammatory factor in the formation of atherosclerotic lesions. | [4] |
| IL17RA | The IL17A/IL17RA axis has pro-inflammatory effects during the progression of atherosclerosis. | [5] |
| SLC11A1 | SLC11A1 is differentially expressed between atherosclerosis samples and control samples. | [6] |
| TLR2 | TLR2 promotes the formation of atherosclerotic plaques and enhances atherosclerosis *via* targeting the cells originate from non-bone marrow. | [7] |
| IL1B | IL1B is implicated in atherosclerosis through inducing generation of cytokines and proteolytic enzymes, thus affecting the formation and stability of atheroma. | [8] |
| LYN | CD36 could bind to the oxidized LDL, thus activating LYN by the recruitment of Na/K-ATPase-LYN complex and enhancing the development of atherosclerosis. | [9] |
| CKAP4 | The relationship between CKAP4 and atherosclerosis has not been reported. We assume that CKAP4 may participate in atherosclerosis by regulating the the α5β1 integrin based on previous studies, however, it still needs further investigation. | [10] [11] |

**Reference**

1. Chen Q, Lv J, Yang W, Xu B, Wang Z, Yu Z, Wu J, Yang Y, Han Y: **Targeted inhibition of STAT3 as a potential treatment strategy for atherosclerosis**. *Theranostics* 2019, **9**(22):6424-6442.

2. Dewberry R, Holden H, Crossman D, Francis S: **Interleukin-1 receptor antagonist expression in human endothelial cells and atherosclerosis**. *Arterioscler Thromb Vasc Biol* 2000, **20**(11):2394-2400.

3. An G, Ren G, An F, Zhang C: **Role of C5a-C5aR axis in the development of atherosclerosis**. *Sci China Life Sci* 2014, **57**(8):790-794.

4. Lehrke M, Millington SC, Lefterova M, Cumaranatunge RG, Szapary P, Wilensky R, Rader DJ, Lazar MA, Reilly MP: **CXCL16 is a marker of inflammation, atherosclerosis, and acute coronary syndromes in humans**. *J Am Coll Cardiol* 2007, **49**(4):442-449.

5. Butcher MJ, Gjurich BN, Phillips T, Galkina EV: **The IL-17A/IL-17RA axis plays a proatherogenic role via the regulation of aortic myeloid cell recruitment**. *Circ Res* 2012, **110**(5):675-687.

6. Hagg DA, Jernas M, Wiklund O, Thelle DS, Fagerberg B, Eriksson P, Hamsten A, Olsson B, Carlsson B, Carlsson LM *et al*: **Expression profiling of macrophages from subjects with atherosclerosis to identify novel susceptibility genes**. *Int J Mol Med* 2008, **21**(6):697-704.

7. Roshan MH, Tambo A, Pace NP: **The Role of TLR2, TLR4, and TLR9 in the Pathogenesis of Atherosclerosis**. *Int J Inflam* 2016, **2016**:1532832.

8. Bhaskar V, Yin J, Mirza AM, Phan D, Vanegas S, Issafras H, Michelson K, Hunter JJ, Kantak SS: **Monoclonal antibodies targeting IL-1 beta reduce biomarkers of atherosclerosis in vitro and inhibit atherosclerotic plaque formation in Apolipoprotein E-deficient mice**. *Atherosclerosis* 2011, **216**(2):313-320.

9. Uchiumi F, Semba K, Yamanashi Y, Fujisawa J, Yoshida M, Inoue K, Toyoshima K, Yamamoto T: **Characterization of the promoter region of the src family gene lyn and its trans activation by human T-cell leukemia virus type I-encoded p40tax**. *Mol Cell Biol* 1992, **12**(9):3784-3795.

10. Osugi Y, Fumoto K, Kikuchi A: **CKAP4 Regulates Cell Migration via the Interaction with and Recycling of Integrin**. *Mol Cell Biol* 2019, **39**(16).

11. Al-Fakhri N, Wilhelm J, Hahn M, Heidt M, Hehrlein FW, Endisch AM, Hupp T, Cherian SM, Bobryshev YV, Lord RS *et al*: **Increased expression of disintegrin-metalloproteinases ADAM-15 and ADAM-9 following upregulation of integrins alpha5beta1 and alphavbeta3 in atherosclerosis**. *J Cell Biochem* 2003, **89**(4):808-823.
